# Travelling-wave gel dipolophoresis of hydrophobic conducting colloids

**DOI:** 10.1140/epje/s10189-025-00492-5

**Published:** 2025-05-24

**Authors:** Touvia Miloh, Eldad J. Avital

**Affiliations:** 1 School of Mechanical Engineering, Tel Aviv University, 69978 Tel-Aviv, Israel; 2 School of Engineering and Materials Science, Queen Mary University of London, London, E1 4NS UK

## Abstract

A unified ‘weak-field’ formulation is provided for calculating the combined nonlinear effect of dielectrophoresis and the induced-charge electrophoresis (dipolophoresis) of polarized rigid hydrophobic spherical colloids freely suspended in an electrolyte-saturated Brinkman-hydrogel (porous) medium under a general (direct or alternating currents) non-uniform electric forcing. Explicit expressions for the modified total dipolophoretic mobility of a conducting (metallic) spherical colloid are given in terms of the Brinkman (Darcy), Navier slip, and Debye (screening) length scales. Also presented is a rigorous derivation of the Helmholtz–Smoluchowski slip velocity in terms of these three length scales, including the induced electroosmotic flow field around a hydrophobic rigid colloid embedded in a Brinkman medium that is forced by an arbitrary (non-uniform) ambient electric field. The available solutions for a free (non-porous) electrolyte solution under a uniform forcing and no-slip surface are obtained as limiting cases. For the purpose of illustration, we present and analyse some newly explicit solutions for the mobility and the associated induced-charge electroosmotic velocity field of a slipping colloid set in an effective (hydrogel) porous medium, which is exposed to an ambient ‘sinusoidal’ travelling-wave excitation depending on frequency and wave number.

**Graphical abstract:**

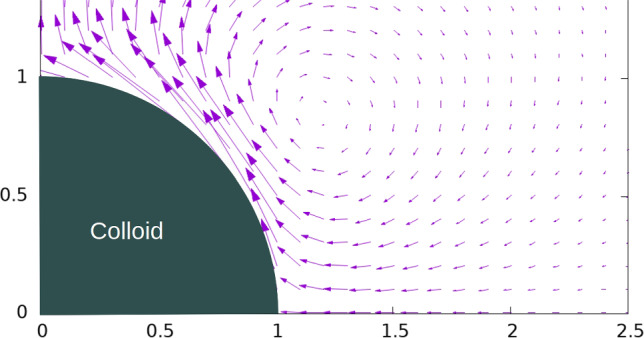

## Introduction

Gel electrophoresis (GE) is a pervasive technique used for separating and analysing bio-macromolecules and nanoparticles such as DNA, RNA, and proteins, based on size and shape by means of applying electric fields in various microfluidic devices [1–8]. The surrounding hydrogel medium is usually made of a mesh of polyacrylamides of different sizes, which can be modelled as an effective porous (polymeric) medium depending on a single Darcy–like coefficient characterizing the ratio between the particle size to that of the average gel pores, by using the prevalent Brinkman-Debye-Bueche (BDB) Stokes linear model [9, 10]. Most GE studies consider uniformly charged dielectric colloids (constant zeta potential) embedded in a gel matrix with prescribed (either mobile or immobile) charge densities that are forced by applying a uniform direct current (DC) electric signal over a non-slip or slipping (hydrophobic) colloidal surfaces [11, 12] or for example a constant concentration gradient in the case of diffusiophoresis [13, 14].

Electrophoresis is generally associated with the motion of freely suspended charged particles through a liquid medium by applying an ambient uniform electric field in a microchannel bounded by electrodes. The resulting colloid mobility due to the Coulomb force in DC scenarios is linear in the applied field and the ambient surface charge (zeta potential) of the particle [15] and thus a modified (Henry type) formula for the steady mobility of a charged colloid moving in a gel (porous) medium can be obtained in terms of the Brinkman parameter [3] through linearization. In a similar manner, an explicit solution for the time-depended mobility of a weakly charged colloid suspended in hydrogel resulting from the transient electroosmotic flow incited by a suddenly applied (Heaviside) uniform field can be obtained as a function of the screening electric double layer (EDL) and the ambient charges in the gel matrix [16]. Nevertheless, it should be mentioned that these mobility (electrophoretic) studies, related to uniformly charged colloids and fixed gel charges, apply only to a uniform DC electric forcing. Thus, under alternating-current (AC) excitation and due to the linear dependence of the mobility on the field, the colloid velocity averaged over a period is null, unless nonlinear effects due to polarization are considered which results in a quadratic dependence of the mobility on the amplitude of the field [17].

If the colloids are conducting (metallic) and initially uncharged, they can still acquire a finite mobility (both under direct or alternating currents excitation) due to the ‘induced-charge electro-osmotic’ (ICEO) velocity field generated around the free particle and the interaction between the ambient electric field and the ionic charge distribution incited by the Poisson equation around the polarizable particle [18, 19]. The resulting induced-charge electrophoretic (ICEP) mobility of the conducting colloid is quadratic in the amplitude of the ambient field (Coulomb’s law) and thus one can get a non-vanishing mobility even under an oscillatory (AC) excitation. When the applied electric field is spatially *non-uniform*, there is another propulsion mechanism in addition to ICEP due to dielectrophoresis (DEP), depending on the field gradients and higher-order field derivatives [20]. The resulting total dipolophoretic (DIP) velocity of the free colloid is thus defined by the combination of both DEP and ICEP effects. The term ‘dipolophoresis’ was probably first coined by the Russian school [21] and has been since extensively used in the literature in the context of related problems [22–29].

As demonstrated in the above studies, the general DIP formulation is essentially nonlinear; however, it is still possible to obtain some simple practical analytical expressions for the mobility of spherical uncharged polarized particles freely suspended in non-uniform DC/AC fields, by invoking the ‘weak-field’ assumption and consequently through the process of linearizing the Poisson–Nernst–Planck (PNP) system of equations [30, 31]. In addition, by applying the Teubner integral approach [32] (valid for unrestricted EDLs), one can get explicit expressions for the ICEP force exerted on the initially uncharged polarizable particle. The corresponding DEP force acting on a rigid (non-deformable) spherical colloid can be found by the method of multipole expansions [22–24] which together with ICEP determines the DIP force and consequently the colloid mobility. The hydrodynamic model in a free electrolyte (clean) solution (i.e. non-porous medium) employed in the original Teubner’s formulation [32] is based on using the singularity method [33] for the steady Stokes equation (creeping motion) of a homogeneous liquid medium and rigid particles, under the common no-penetration and no-slip velocity boundary conditions [34].

One of the main goals of the present study is to extend the above electrophoretic (linear in the field) methodologies for determining the DIP (quadratic in the field) mobility of a hydrophobic (slipping) initially uncharged polarized spherical colloid embedded in an unbounded Stokes-Brinkman porous medium under any DC/AC non-uniform electric forcing while preserving the surface Navier slip boundary condition [35, 36]. It should be noted that this is an intricate case of dielectrophoresis (DEP) and induced-charge electroosmosis (ICEO) over ideally polarizable (conducting) particles. In such cases, the equilibrium zeta potential on the colloid and the fixed (immobile) charges in the gel can be ignored with respect to the induced charges governed by the Poisson equation, see for example [3–5, 17–19].

We provide new analytic solutions for the DIP phoretic velocity of a slipping sphere embedded in a porous medium, in terms of the Brinkman, Navier slip and Debye (EDL) length scales, thus generalizing the existing ICEP studies [17–32] for free (non-porous) electrolyte solutions and non-slipping colloids. Simplified expressions for the colloid motility and the spatial ICEO velocity field around the particle are provided by assuming a thin EDL (small Debye scale), although the general formulation holds for unrestricted EDLs [22]. Rigorous expressions based on Teubner’s formulation [32] are also derived for the modified Helmholtz–Smoluchowski (HS) slip velocity on the colloid surface in a gel and are compared against the available heuristic approximations [37–40] for a non-porous medium. Finally, we consider the intricate case of non-uniform (DC/AC) electric excitations, including the special case of a ‘sinusoidal’ travelling-wave electrophoresis (TWEP) [41–49], where the mobility of a polarizable hydrophobic spherical colloid moving in a gel (porous) solute is explicitly expressed in terms of the amplitudes, wave number and frequency of the time-harmonic wave forcing. In addition, we provide analytic solutions for the nonlinear ICEO velocity field (stream function) prevailing around a stationary hydrophobic colloid embedded in a hydrogel that is subject to a ‘sinusoidal’ or any DC/AC non-uniform electric signal. Limiting cases, corresponding to non-porous free medium, non-slipping surface, time-independent (DC), and uniform forcing, are also discussed.

The structure of the paper is as follows: In Sect. 2, we formulate the electrostatic problem which is uncoupled from the hydrodynamic problem by ignoring (small Peclet number) the convection term with respect to the diffusion and electro-migration terms [17] and linearizing the PNP set of equations under the ‘weak-field’ assumption [31]. Then, we consider an arbitrary (DC/AC) ambient spatially non-uniform electric field (preserving Laplace’s equation) expressed as a series of Legendre polynomials with prescribed amplitudes and provide an explicit general expression for the DEP force exerted on a free polarized spherical particle, using the multipole methodology outlined in [23]. The hydrodynamic problem presented in Sect. 3 is formulated as a linearized non-homogeneous Stokes-Brinkman momentum equation (Newtonian fluid and incompressible flow), modelling an effective porous medium that is forced by the (quadratic) Columbic term and a Navier slipping surface [16, 35]. By employing Teubner’s [32] integral formulation, the corresponding ICEO force acting on the polarized colloid is obtained in terms of the three characteristic length scales, namely the Brinkman (Darcy), Navier slip and the Debye (EDL).

Under the assumption of a thin EDL, we rigorously derive in Sect. 4 an explicit expression for the generalized HS slip velocity prevailing on the surface of a conducting hydrophobic particle freely suspended in a Brinkman saturated electrolyte, which reduces under the proper limits to the existing expression for the ordinary free electrolyte solution of a slipping surface [37]. The explicit solutions thus found for the DEP and ICEP forces exerted on a slipping colloid embedded in a Brinkman solute under a general non-uniform electric forcing are then combined in Sect. 5 in order to find new expressions for the total DIP force acting on an hydrophobic spherical colloid, as well as for the Stokes stream function of the resulting ICEO velocity field around it. In addition, we provide a new analytical solution for the mobility of a free hydrophobic particle moving in an hydrogel under a sinusoidal travelling-wave (TW) excitation, depending on the frequency and wave number of the ambient electric signal, generated for example by using interdigitated electrode arrays [50]. Finally, we present a detailed solution for the quadrupolar [17] ICEO flow field (Stokes stream function) incited around a Navier slipping spherical colloid lying in a saturated Brinkman-hydrogel medium under any non-uniform DC/AC forcing, expressed in terms of Legendre polynomials, including a simple DC sine-wave signal. We conclude with a discussion of the new DIP solutions thus found and the numerical simulations, including a comparison of the limiting cases (i.e. non-porous solute, no-slip surface, DC vs. AC excitation as well as non-uniform vs. uniform forcing) against existing solutions, followed by a summary of the main results deduced in this study.

## The electrostatic problem and DEP force

We consider an initially uncharged conducting (ideally polarized) hydrophobic (slipping) spherical colloid of radius *a*, which is freely suspended in an unbounded gel electrolyte and is subjected to an arbitrary spatially non-uniform ambient DC or AC electric field of frequency $\omega$. The hydrogel (porous medium) binary electrolyte is z-z symmetric with constant diffusivity D and the surface of the inert particle is considered as impermeable to ions. The problem is formulated in terms of a spherical coordinate system $\left( {R,\theta ,\varphi } \right)$ centred at the colloid, such that the travelling-wave (TW) electric forcing propagates along $\theta = 0.$ Accordingly, one can also define an inertial Cartesian system (*x*_1_, *x*_2_, *x*_3_), where $x_{1} = R*\mu , x_{2} + ix_{3} = R\left( {1 - \mu^{2} } \right)^{1/2} e^{i\varphi }$ and $\mu = \cos \theta$, such that *x*_1_ coincides with $\theta = 0$. Taking advantage of the azimuthal symmetry with respect to the *x*_1_ axis, the dimensionless (using a reference amplitude *E*_0_) ambient non-uniform AC electric field which is represented here in terms of its phasor is given by $\tilde{\chi }_{am} \left( {r,\mu ,t} \right) = {\text{Re}}\left\{ {\chi_{am} \left( {r,\mu } \right)e^{ - i\omega t} } \right\}$, where Re denotes the real part and *r* = *R*/a (non-dimensional). Note that both $\tilde{\chi }_{am}$ and $\chi_{am}$ are harmonic (axisymmetric) functions satisfying Laplace’s equation. Similarly, one can also define the dimensionless electric potential $\tilde{\phi }\left( {r,\mu ,t} \right)$ (scaled by $E_{0} a$) and the induced-charge distribution in the electrolyte $\tilde{Q}\left( {r,\mu ,t} \right)$ (scaled following the Poisson equation by $\epsilon E_{0} /a$ where $\epsilon$ is the solute permittivity) in terms of their phasors $\phi \left( {r,\mu } \right)$ and $Q\left( {r,\mu } \right)$, using the common normalization with respect to the thermal scale [23].

Analytic solutions of the electrostatic problem can be obtained by invoking the so-called ‘weak field’ assumption [30, 31] and ignoring convection effects with respect to electro-migration and diffusion (small Peclet) [15], which divulges the uncoupling between the electrostatic and the hydrodynamic problems. Thus, by employing a small parameter defined by the ratio of the characteristic amplitude *E*_0_ of the ambient field and the thermal scale (potential), the corresponding Poisson-Nernst-Planck (PNP) system of equations can be linearized with respect to the same small parameter. Following the above procedure, the normalized phasors of the potential and induced charge are related by [23];

1 $$ 2\lambda_{0}^{2} \nabla^{2} \phi = - \lambda^{2} \nabla^{2} Q = - Q,\quad \quad \quad \frac{1}{{\lambda^{2} }} = \frac{1}{{\lambda_{0}^{2} }} - \frac{{i\omega a^{2} }}{D}, $$

where $\lambda_{0} \left( {{\text{real}}} \right)$ denotes the dimensionless Debye (EDL) thickness [15] and $\lambda$ defined in Eq. (1) is a frequency-dependent complex parameter. Ignoring the effect of surface conductance (small Dukhin number) and supplementing Eq. (1) are the no-ion penetration and zero potential boundary conditions prevailing over the conducting surface [24], i.e.

2 $$ 2\frac{\partial \phi }{{\partial r}} + \frac{\partial Q}{{\partial r}} = 0; \quad \quad \phi = 0\quad \quad {\text{on }}r = 1, $$

together with the appropriate far-field decay requirement, namely $\phi \to \chi_{am}$ and $Q \to 0$ for $r \to \infty$. Following Eq. (1), it is thus possible to express the electric potential as

3 $$ 2\phi = - \left( {\frac{\lambda }{{\lambda_{0} }}} \right)^{2} Q + \chi , \quad \quad \quad \nabla^{2} \chi = 0, $$

where the disturbance potential $\chi \left( {r,\mu } \right)$ is an arbitrary general (complex) harmonic function, which can be written by virtue of the above as

4 $$ \chi \left( {r,\mu } \right) = - - 2\mathop \sum \limits_{n = 1}^{\infty } \left[ {A_{n} r^{n} + B_{n} r^{{ - \left( {n + 1} \right)}} } \right]P_{n} \left( \mu \right),\quad \quad \chi_{am} \left( {r,\mu } \right) = - \mathop \sum \limits_{n = 1}^{\infty } A_{n} r^{n} P_{n} \left( \mu \right). $$

where for simplicity (excluding a constant value) we assume that the ambient potential vanishes at the origin ($r = 0$). Here, $P_{n} \left( \mu \right)$ represent the common Legendre polynomials of order n and the coefficients *B*_*n*_ have to be determined in terms of the prescribed amplitudes *A*_*n*_ of the applied field $\chi_{am}$.

A general solution for the induced-charge distribution can be found by solving the corresponding Helmholtz equation (Eq. 1), providing [23]

5 $$ \begin{aligned}& Q\left( {r,\mu } \right) = - 2\mathop \sum \limits_{n = 1}^{\infty } C_{n} K_{n} \left( {r/\lambda } \right)P_{n} \left( \mu \right), \quad  \\ &K_{n} \left( {r/\lambda } \right) = \frac{{e^{{\left( {1 - r} \right)/\lambda }} }}{r}\frac{{\mathop \sum \nolimits_{m = 0}^{n} \left( {n + 1/2,m} \right)\left( {\frac{\lambda }{2r}} \right)^{m} }}{{\mathop \sum \nolimits_{m = 0}^{n} \left( {n + 1/2,m} \right)\left( {\frac{\lambda }{2}} \right)^{m} }}, \end{aligned}$$

where $\left( {n + 1/2,m} \right) = \left( {n + m} \right)!/\left[ {m!\left( {n - m} \right)!} \right]$ and $K_{n} \left( {1/\lambda } \right) = 1$. Finally, imposing the surface boundary conditions of Eq. (2) renders the following explicit expressions for the coefficients $\left( {B_{n} ,C_{n} } \right)$ in terms of $A_{n}$;

6 $$\begin{aligned} &B_{n} = \frac{{n - i\Omega_{n} }}{{n + 1 + i\Omega_{n} }}A_{n} , \\ & C_{n} = \frac{2n + 1}{{n + 1 + i\Omega_{n} }}\left( {\frac{{\lambda_{0} }}{\lambda }} \right)^{2} A_{n} , \\ & \Omega_{n} = \frac{{\omega a\lambda_{0}^{2} }}{D}\dot{K}_{n} ,\end{aligned} $$

where $\dot{K}_{n} = \frac{{\text{d}}}{{{\text{d}}r}}K_{n} \left( {r/a} \right)|_{r = 1}$ and according to Eqs. (1) and (5), one readily finds that $\dot{K}_{n} \simeq - 1/\lambda_{0}$ as $\lambda_{0} \to 0$(thin EDL). Once an explicit solution for the electrostatic problem is found (see Eqs. (2–6)), one can readily determine the time-average dielectrophoretic (DEP) force acting on the freely suspended spherical colloid in terms of the ambient (forcing) field coefficients $A_{n}$ following the procedure outlined in [22–24]. Thus, given the inhomogeneous electrostatic potential in Eq. (4), the resulting DEP force exerted on the free colloid along the *x*_1_ axis can be explicitly written in terms of the coefficients *A*_n_ of the ambient field ( see Eq. (4)) and the corresponding multipoles *B*_n_ by applying the following relation involving the Legendre polynomials [51, 52]

7 $$ r^{{ - \left( {n + 1} \right)}} P_{n} \left( \mu \right) = \frac{{\left( { - 1} \right)^{n} }}{{n{!}}}\frac{{\partial^{n} }}{{\partial x_{1}^{n} }}\left( \frac{1}{r} \right), \quad \quad \lim_{r \to 0} \frac{{\partial^{n} }}{{\partial x_{1}^{n} }}\{ r^{m} P_{m} (\mu )\} = n!\delta (n - m) $$

which together with Eq. (6) and following the methodology of [23, 24], finally renders for $r \to 0$;

8 $$ F_{{{\text{DEP}}}}^{\left( 1 \right)} = - 2\pi \mathop \sum \limits_{n = 1}^{\infty } \mathop \sum \limits_{m = 1}^{\infty } \frac{{A_{m} B_{n}^{*} }}{{n{!}}}\frac{{\partial^{n + 1} }}{{\partial x_{1}^{n + 1} }}\left( \frac{1}{r} \right) = - 2\pi \mathop \sum \limits_{n = 1}^{\infty } \left( {n + 1} \right)A_{n}^{*} A_{n + 1} \frac{{n + i\Omega_{n} }}{{n + 1 - i\Omega_{n} }}, $$

where the superscript (*) denotes a complex conjugate. Equation (8) provides a useful non-dimensional (with respect to $\epsilon E_{0}^{2} a^{2}$) analytic expression for the DEP force exerted on a free spherical colloid in terms of the prescribed forcing amplitudes $A_{n}$ and the generalized RC frequencies $\Omega_{n}$ defined in Eq. (6). In the DC limit $\left( {\omega = 0} \right)$, one gets from Eq. (6) $\Omega_{n} = 0$ and Eq. (8) reduces to the DEP expression $- 4\pi \mathop \sum \nolimits_{n = 1}^{\infty } nA_{n} A_{n + 1}$ given in [20], providing the coefficients *A*_n_’s are all real. Note also the difference by a factor of 2 between the above DC and AC formulations due to the time-averaging procedure [24].

## The hydrodynamic problem and ICEP force

It has been demonstrated that gel-electrophoresis in a polymeric (porous) medium can be modelled by the inhomogeneous Stokes-Brinkman momentum equation of an incompressible medium forced by the Columbic force, leading to the following fluid transport equations [36];

9 $$ \nabla P = \nabla^{2} {\varvec{u}} - \alpha^{2} {\varvec{u}} - \frac{1}{{2\lambda_{0}^{2} }}Q^{*} \nabla \phi ,\quad \quad \nabla \cdot {\varvec{u}} = 0 $$

where *P* and ${\mathbf{u}}$ denote the dimensionless time-average pressure and velocity field, and $\alpha = 1/\sqrt {K_{p} }$ is the frictional dimensionless coefficient defined in terms of the Darcy permeability *K*_*p*_ parameter [53, 54]. One should also mention here the close analogy between the linearized unsteady Stokes equation and the Brinkman formulation [55], which is evidenced and discussed in some of the recent publications on the subject [16, 35, 56–61]. In addition, it is worth noting that the colloid itself can also be considered as a porous medium (polyelectrolyte) so that in the more general case, both the surrounding hydrogel matrix and the colloid are treated as effective porous media with distinct Brinkman (Darcy) coefficients [62].

Closure of the governing non-dimensional hydrodynamic equations is provided by applying the Navier–Maxwell–Basset slip boundary condition ( surface velocity slip proportional to the tangential viscous stress component) prevailing on the surface of the impermeable hydrophobic spherical colloid translating with a constant veloci**t**y ${\mathbf{U}}$[34, 35], i.e.

10 $$ {\varvec{u}} = {\varvec{U}} + \beta \left( {{\varvec{\sigma}} \cdot {\varvec{n}}} \right) \cdot \left( {{\varvec{I}} - {\varvec{nn}}} \right)\quad {\text{on}}\quad r = 1, $$

where $\beta$ denotes the dimensionless Navier slip coefficient (scaled with respect to $a/\eta$ with $\eta$ representing the dynamic viscosity), ranging from zero for no-slip to infinity for a perfect slip [34]. In addition, ***I*** is the unitary matrix, ${\mathbf{n}}$ denotes the outward unit vector normal to the spherical surface and ${\varvec{\sigma}}$ represents the corresponding time-average hydrodynamic stress, such that in lieu of Eq. (9) one gets

11 $$ \nabla \cdot {\varvec{\sigma}} = \alpha^{2} {\varvec{u}} + \frac{1}{{4\lambda_{0}^{2} }}Q^{*} \nabla \chi . $$

Note that following Eq. (3), the Columbic forcing term in Eq. (9) can be split into a gradient term and $Q^{{*}{}} \nabla \chi /(4\lambda_{0}^{2} )$. The remaining gradient term which is proportional to $\nabla \left| {Q^{2} } \right|$ can then be included in the hydrodynamic pressure term which results in Eq. (11).

In order to determine the hydrodynamic ICEP force ${\varvec{f}}_{{\varvec{H}}} = \mathop \int \limits_{S} {\varvec{\sigma}} \cdot {\varvec{n}}{\text{d}}s$ exerted on the colloid, we make use of the Lorentz reciprocal theorem [35, 63] by defining an auxiliary problem governed by the homogenous associate (unforced) Brinkman equation

12 $$ \nabla \hat{P}^{\left( i \right)} = \nabla^{2} \hat{\user2{u}}^{{\left( {\varvec{i}} \right)}} - \alpha^{2} \hat{\user2{u}}^{{\left( {\varvec{i}} \right)}} , \quad \quad \nabla \cdot \hat{\user2{u}}^{{\left( {\varvec{i}} \right)}} = 0, $$

such that $\nabla \cdot \hat{\user2{\sigma }}^{{\left( {\varvec{i}} \right)}} = \alpha^{2} \hat{\user2{u}}^{{\left( {\varvec{i}} \right)}}$ and the auxiliary velocity field $\hat{\user2{u}}^{{\left( {\varvec{i}} \right)}}$ denotes the velocity field induced in the hydrogel solute by a colloid translating with a unit velocity along the $x_{i}$ axis and is subject on *r* = 1 to

13 $$ \hat{\user2{u}}^{{\left( {\varvec{i}} \right)}} = {\varvec{I}} + \beta \left( {\hat{\user2{\sigma }}^{{\left( {\varvec{i}} \right)}} \cdot {\varvec{n}}} \right) \cdot \left( {{\varvec{I}} - {\varvec{nn}}} \right), $$

where $\hat{\user2{\sigma }}^{{\left( {\varvec{i}} \right)}}$ is the corresponding viscous shear stress of the auxiliary problem governed by Eq. (12) (see [32]).

Applying the reciprocal theorem to the two related hydrodynamic problems $({\mathbf{u}},{\boldsymbol{\sigma }})$ and $({\hat{\mathbf{u}}}^{{{\mathbf{(i)}}}} ,{\hat{\boldsymbol{\sigma }}}^{{{\mathbf{(i)}}}} )$ implies that14where  denotes the fluid volume (unbounded) exterior to the particle and $S$ represents the surface of the colloid.

Next, focusing our interest on the resultant force acting on the colloid and hence the surface integral of the time-average hydrodynamic stress over the colloid, Eq. (11) substituted along with $\nabla \hat{\user2{\sigma }}^{{\left( {\varvec{i}} \right)}} = \alpha^{2} \hat{\user2{u}}^{{\left( {\varvec{i}} \right)}}$ in Eq. (13) renders15where $F_{H}^{\left( i \right)}$ denotes the component of the hydrodynamic force acting along the $x_{i}$ direction. In addition to this hydrodynamic (ICEP) force resulting from the induced-charge electroosmotic velocity field, the free colloid is also subject to an electrostatic (Coulomb’s law) force, given by $F_{E}^{(i)} = - \frac{1}{{4\lambda_{0}^{2} }}\int_{\forall } {Q^{*} } \frac{\partial \chi }{{\partial x_{i} }}{\text{d}}\forall$ due to the non-homogenous (forcing) term on the right-hand side of Eq. (9). Finally, since the particle is force-free, namely $F_{H}^{\left( i \right)} + F_{E}^{\left( i \right)} = 0$, one gets from Eq. (15), an explicit (real) expression for the colloid combined mobility *U*_i_, i.e. the colloid’s transport equation [22, 32];16where $\mathop \int \limits_{S} \sigma_{jk}^{\left( i \right)} n_{k} {\text{d}}s = R^{T} \delta_{ij}$ and $R^{T} (\alpha ,\beta )$ represents the dimensionless resistance coefficient of a translating slipping sphere in a Brinkman medium [64]. As shown in the Appendix, the generalized translation-resistance coefficient for a Navier slipping hydrophobic sphere embedded in a Brinkman fluid depends on the two length scales $(\alpha ,\beta )$ and can be simply written as [35];

17 $$ R^{T} \left( {\alpha ,\beta } \right) = - 6\pi \left[ {\frac{{\left( {1 + \alpha } \right)\left( {1 + 2\beta } \right)}}{{1 + \beta \left( {3 + \alpha } \right)}} + \frac{{\alpha^{2} }}{9}} \right], $$

Equation (17) reduces to the well-known limiting solutions, namely $R^{T} \left( {0,0} \right) = - 6\pi$ (Stokes no-slip), $R^{T} \left( {0,\beta } \right) = - 6\pi \left( {1 + 2\beta } \right)/\left( {1 + 3\beta } \right)$ (Stokes Navier slip) [33] and $R^{T} (\alpha ,0) = - 6\pi (1 + \alpha + \alpha^{2} /9)$ (Brinkman no-slip) [35, 55]. Note that the last expression corresponds to the drag force experienced by a steadily moving no-slip colloid in a Brinkman fluid and is different from the drag exerted on a stationary colloid placed in a uniform stream as discussed in [9, 53, 65]. Thus, instead of the quadratic term $\alpha^{2} /9$ in Eq. (17), an equivalent term $\alpha^{2} /3$ appears in the original work by Brinkman [9] which also corresponds to the frequency-dependent force acting on an oscillating sphere in the same liquid medium [33, 66]. It is also worth mentioning here the similarity between steady Brinkman flows under a Navier slip, with transient or oscillatory slipping Stokes flows, especially when the slip parameter $\beta$ is relatively large [57].

## Thin EDL

The Teubner integral derived in Eq. (16) is exact in the sense that it applies to an arbitrary Debye scale (unrestricted EDL thickness), as well as for a hydrophobic (slipping) spherical colloid embedded in a porous (Brinkman) medium. In the limit of $\alpha = 0$ (non-porous ‘clean’ electrolyte) and $\beta = 0$ (no-slip) impermeable particles, it renders as demonstrated in [22–24] the dipolopheretic mobility of a free rigid polarizable spherical particle which is forced by either DC or AC electric fields. The volume integral in Eq. (16) can be further simplified under the assumption of an asymptotically thin EDL, by recalling in lieu of Eq. (5) that the induced-charge distribution in the solute decays exponentially away from the colloid as $e^{{ - \left( {r - 1} \right)/\lambda }}$. Thus, we note using integration by parts that as $\lambda \to 0 ;$

18 $$ \mathop \int \limits_{1}^{\infty } G\left( r \right)e^{{ - \left( {r - 1} \right)/\lambda }} {\text{d}}r \sim \lambda G\left( 1 \right) + \lambda^{2} G^{\prime } \left( 1 \right) + \lambda^{3} G^{\prime \prime } \left( 1 \right) + \cdots , $$

where *G*(*r*) represents a well-behaved differentiable function and the prime denotes differentiation with respect to the argument (radius).

Applying Eq. (18) to the ICEP (Teubner) integral in Eq. (16) and assuming thin EDL implies that to leading order in $\lambda$

19 $$ U_{i} R^{T} = \frac{1}{{4\lambda_{0}^{2} }}\mathop \int \limits_{S} \mathop \int \limits_{1}^{\infty } \left( {\hat{u}_{j}^{\left( i \right)} - \delta_{ij} } \right)Q_{0}^{*} \left( {r,\mu } \right)\frac{\partial \chi }{{\partial x_{j} }}e^{{ - \left( {r - 1} \right)/\lambda }} {\text{d}}r{\text{d}}S, $$

where we define $Q\left( {r,\mu } \right) = Q_{0} \left( {r,\mu } \right)e^{{ - \left( {r - 1} \right)/\lambda }}$ (see Eq. (5)) and *S* denotes the wetted surface of the free colloid. For a thin EDL and moderate frequencies well below the Maxwell–Wagner (MW) limit (i.e. $\omega \lambda_{0}^{2} /D \ll 1),$ $\lambda$ can be replaced by $\lambda_{0}$ and considered as a real parameter (see Eq. (1). In addition, we recall that due to the time-averaging procedure over a single period, the factor 4 in Eq. (19) obtained for AC forcing should be replaced by 2 for DC excitations [23]. Thus, the formulation in the sequel holds for both DC and AC electric forcing but they differ by a factor of 2.

Substituting the two-term thin EDL approximation of Eq. (18) in Eq. (19) renders

20 $$ \begin{aligned} U_{i} R^{T} & = \frac{1}{{4\lambda_{0} }}\mathop \int \limits_{S} \left( {\hat{u}_{j}^{\left( i \right)} - \delta_{ij} } \right)Q_{0}^{*} \left( {1,\mu } \right)\frac{\partial \chi }{{\partial x_{j} }}{\text{d}}S \\ & \quad + \frac{1}{4}\mathop \int \limits_{S} \frac{{\partial \hat{u}_{j}^{\left( i \right)} }}{\partial r}Q_{0}^{*} \left( {1,\mu } \right)\frac{\partial \chi }{{\partial x_{j} }}{\text{d}}S \\ & \quad  + \frac{1}{4}\mathop \int \limits_{S} \left( {\hat{u}_{j}^{\left( i \right)} - \delta_{ij} } \right)Q_{0}^{*} \left( {1,\mu } \right)\frac{{\partial^{2} \chi }}{{\partial r\partial x_{j} }}{\text{d}}S\\ & \quad  + O\left( {\lambda_{0} } \right)\quad {\text{on}}\quad r = 1. \\ \end{aligned} $$

Note that under a DC forcing $(\omega = 0)$, Eqs. (1–3) imply that $\partial \chi /\partial r = 0$ on $S$ , and for this reason, the third integral in Eq. (20) vanishes. Furthermore, for AC excitation (thin EDL), one gets [24] $\partial \chi /\partial r = - (i\omega a\lambda_{0} /D)Q$ on $S$, which indicates that the third integral is purely imaginary and thus can be neglected in the present context. Finally, by enforcing the Navier slip boundary condition on $S$ (*r* = 1) from Eq. (13), one gets the following expression (correct to leading-order in both $\beta$ and $\lambda_{0}$) for the mobility of the hydrophobic colloid

21 $$\begin{aligned} U_{i} R^{T} &\approx \frac{1}{4}\mathop \int \limits_{S} \left[ {\frac{{\partial \hat{u}_{j}^{\left( i \right)} }}{\partial r} + \frac{\beta }{{\lambda_{0} }}\left( {\delta_{jk} - n_{j} n_{k} } \right)\hat{\sigma }_{km}^{\left( i \right)} n_{m} } \right]\nonumber \\
& \quad Q_{0}^{*} \left( {1,\mu } \right)\frac{\partial \chi }{{\partial x_{j} }}{\text{d}}S{,}\quad {\text{on}}\quad {\text{r}} = 1.\end{aligned} $$

The solution of the auxiliary hydrodynamic problem $\left( {\hat{u}_{j}^{\left( i \right)} ,\hat{\sigma }_{jk}^{\left( i \right)} } \right)$ is detailed in the Appendix, where explicit expressions for the surface traction $\hat{\sigma }_{jk}^{\left( i \right)} n_{k}$ and $\partial \hat{u}_{j}^{\left( i \right)} /\partial r$ evaluated on $S$ are presented in Eqs. (75) and (79) in terms of the coefficients $K,L,M,N,G_{1}$ and $G_{2}$(see Appendix). Thus, the term in the square parenthesis of Eq. (21) can be written as

22 $$ \begin{aligned} &\frac{{\partial \hat{u}^{{\left( i \right)}} }}{{\partial r}} + \frac{\beta }{{\lambda _{0} }}\left( {\delta _{{jk}}  - n_{j} n_{k} } \right)\left( {\hat{\sigma }_{{km}}^{{\left( i \right)}} n_{m} } \right)\\ &\quad  = \delta _{{ij}} \left\{ G_{1} \left( {\alpha ,\beta } \right) + \frac{\beta }{{\lambda _{0} }}\left[ {b\left( {\alpha ,\beta } \right)K\left( \alpha  \right) + d\left( {\alpha ,\beta } \right)M\left( \alpha  \right)} \right] \right\} \\ &\qquad + n_{i} n_{j} \Big\{ G_{2} \left( {\alpha ,\beta } \right) - \frac{\beta }{{\lambda _{0} }}[ b\left( {\alpha ,\beta } \right)\left( {2L\left( \alpha  \right) + K\left( \alpha  \right)} \right) \\ & \qquad + d\left( {\alpha ,\beta } \right)\left( {2N\left( \alpha  \right) + M\left( \alpha  \right)} \right)]\Big\}.\end{aligned}$$

where the coefficients $b\left( {\alpha ,\beta } \right)$ and $d\left( {\alpha ,\beta } \right)$ are explicitly given in Eq. (77).

To evaluate the resulting mobility of a slipping colloid, let us first note that due to the azimuthal symmetry of $\chi \left( {r,\mu } \right),$ one gets

23 $$ \left( {p\delta_{ij} + qn_{i} n_{j} } \right)\frac{\partial \chi }{{\partial x_{j} }} = p\frac{\partial \chi }{{\partial x_{i} }} + qn_{i} \frac{\partial \chi }{{\partial r}} = n_{i} \left( {p + q} \right)\frac{\partial \chi }{{\partial r}} + \frac{\partial \mu }{{\partial x_{i} }}\frac{\partial \chi }{{\partial \mu }}. $$

where the parameters $p(\alpha ,\beta ,\lambda_{0} )$ and $q\left( {\alpha ,\beta } \right)$ in Eq. (23) are identified as the terms in the curly brackets of Eq. (22) multiplying $\delta_{ij}$ and $n_{i} n_{j}$ , respectively.

Furthermore, to evaluate the surface integral in Eq. (21), we recall following Eq. (3) that the surface of an ideally polarized (conducting) particle can be considered as equipotential $\left( {\phi = 0} \right)$ and thus for thin EDL and moderate frequencies $Q\left( {1,\mu } \right), \simeq \chi \left( {1,\mu } \right)$ and under Eqs. (22) and (23), the kernel in Eq. (21) can be directly written as

24 $$ \chi \left( {p\delta_{ij} + qn_{i} n_{j} } \right)\frac{\partial \chi }{{\partial x_{j} }} = \frac{1}{2}n_{i} \left( {p + q} \right)\frac{{\partial \left| \chi \right|^{2} }}{\partial r} + \frac{p}{2}\frac{\partial \mu }{{\partial x_{i} }}\frac{{\partial \left| \chi \right|^{2} }}{\partial \mu }, $$

where $\partial \mu /\partial x_{1} = - \left( {1 - \mu^{2} } \right)/r$ and $\left( {\partial \mu /\partial x_{2} ,\partial \mu /\partial x_{3} } \right) = - \mu \sqrt {1 - \mu^{2} } \left( {\cos \varphi ,\sin \varphi } \right)/r$. Thus, when integrating over the colloid surface, the first term on the right-hand side of Eq. (24) multiplying $n_{i}$ does not contribute due to asymmetry and the only contribution comes from the second term for *i* = 1, implying that the colloid mobility is directed along the x_1_ axis and is given by

25 $$ U_{1} R^{T} \cong - \frac{\pi }{2}p(\alpha ,\beta ,\lambda_{0} )\mathop \int \limits_{ - 1}^{1} \left( {1 - \mu^{2} } \right)\chi^{*} \left( {1,\mu } \right)\frac{{\partial \chi \left( {1,\mu } \right)}}{\partial \mu }{\text{d}}\mu , $$

where according to Eq. (22):

26 $$ p(\alpha ,\beta ,\lambda_{0} ) = G_{1} \left( {\alpha ,\beta } \right) + \frac{\beta }{{\lambda_{0} }}\left[ {b\left( {\alpha ,\beta } \right)K\left( \alpha \right) + d\left( {\alpha ,\beta } \right)M\left( \alpha \right)} \right]. $$

Note that the coefficients $K\left( \alpha \right)$ and $M\left( \alpha \right)$ depending on the dimensionless Brinkman parameter $\alpha$ are given explicitly in Eq. (75) and $G_{1} \left( {\alpha ,\beta } \right)$ is analytically expressed in Eq. (79).

We recall that under the assumption of a thin EDL, the HS slip velocity for a perfectly conducting no-slip colloid ($\beta = 0$) suspended in a ‘clean’ solution $\alpha = 0$ can be obtained directly by integrating the inhomogeneous Stokes momentum equation (Eq. (9)), resulting in [10, 14] ${\mathbf{u}}_{{\mathbf{s}}} \sim - \frac{1}{4}\chi^{*} \frac{\partial \chi }{{\partial \theta }}{\mathbf{e}}_{{{\varvec{\uptheta}}}}$. Following [67], the mobility of a freely suspended spherical particle moving steadily along the $x_{1}$ axis can then be simply obtained through the generalized Faxen relation as [34, 64];

27 $$ U_{1} = - \frac{1}{2}\mathop \int \limits_{ - 1}^{1} {\varvec{u}}_{s} \cdot {\varvec{e}}_{1} {\text{d}}\mu , $$

where ${\mathbf{e}}_{{\mathbf{1}}}$ and ${\mathbf{e}}_{{{\varvec{\uptheta}}}}$ are two units vectors in Cartesian and spherical coordinates, respectively, such that ${\mathbf{e}}_{{\mathbf{1}}} \cdot {\mathbf{e}}_{{{\varvec{\uptheta}}}} = - \sin \theta$. Note that Eq. (27) can also be interpreted as the average linear velocity taken over the surface of a sphere, and as such is valid for any prescribed slip ${\mathbf{u}}_{{\mathbf{S}}}$. Comparing Eq. (25) with Eq. (27) implies that the modified HS slip velocity in the case of a hydrophobic slipping surface embedded in hydrogel, and can be simply written as

28 $$ {\varvec{u}}_{{\varvec{s}}} = - w\left( {\alpha ,\beta ,\lambda_{0} } \right)\chi^{*} \frac{\partial \chi }{{\partial \theta }}{\varvec{e}}_{{\varvec{\theta}}} ,\quad \quad \quad w\left( {\alpha ,\beta ,\lambda_{0} } \right) = \frac{{\pi p(\alpha ,\beta ,\lambda_{0} )}}{{R^{T} (\alpha ,\beta )}}, $$

which constitutes a generalization of the common HS slip velocity expression for a spherical colloid in the case of a Navier slip $\left( {\beta \ne 0} \right)$ and a porous medium $\left( {\alpha \ne 0} \right)$. It implies that the slip velocity which gives rise to the ICEO flow field around the polarizable particle increases with $\beta$ and decreases with $\alpha$ and $\lambda_{0}$.

Let us next determine the limiting non-porous Stokesian $$\left( {\alpha = 0} \right)$$ value of $$p\left( {0,\beta } \right)$$ under a Navier slip condition $$(\beta \ne 0)$$ [57]. Following Eq. (76), one finds that $$b\left( {0,\beta } \right) = 3\left( {1 + 2\beta } \right)/\left[ {4\left( {1 + 3\beta } \right)} \right]$$ and $$d\left( {0,\beta } \right) = - 3/\left[ {4\left( {1 + 3\beta } \right)} \right]. $$ Recalling that $$A(0) = C(0) = 1$$ (see Appendix), Eq. (79) implies that $$G_{1} \left( {0,\beta } \right) = - 3\left( {2 + 5\beta } \right)/\left[ {4\left( {1 + 3\beta } \right)} \right]$$. Substituting Eq. (75) with the values of *K* (0) = 0 and *M* (0) = 6 in Eq. (26) renders $$p(0,\beta ,\lambda_{0} )$$$$= - 1.5\left[ {\left( {1 + 2.5\beta } \right)/\left( {1 + 3\beta } \right) + \beta /\left( {\lambda_{0} \left( {1 + 3\beta } \right)} \right)} \right]$$ and thus it reduces to the well-known limit (free electrolyte solution Stokes flow and no-slip condition) of $$p(0,0,\lambda_{0} )$$$$= - 3/2$$ [34]. Finally, using this value for $$p(0,\beta ,\lambda_{0} )$$ together with $$R^{T} \left( {0,\beta } \right) = - 6\pi \left( {1 + 2\beta } \right)/\left( {1 + 3\beta } \right)$$ (see Eq. (17)), one gets from Eq. (28);

29 $$ {\varvec{u}}_{{\varvec{s}}} = - \frac{1}{{4\left( {1 + 2\beta } \right)}}\left[ {1 + \frac{5\beta }{2} + \frac{\beta }{{\lambda_{0} }}} \right]\left| \chi \right|\frac{\partial \chi }{{\partial \theta }}{\varvec{e}}_{{\varvec{\theta}}} $$

which for a thin EDL and $\beta /\lambda_{0} = O(1)$, concurs with the corresponding expressions reported in [37–39], suggesting that to leading order in $\beta /\lambda_{0}$, the HS slip for a Navier slipping colloid is proportional to $1 + \beta /\lambda_{0}$. The same dependence can be also obtained in a heuristic manner by noting that $u_{\theta } \sim \beta \frac{{\partial u_{\theta } }}{\partial r}$ on $r = 1$ and using a two-term Taylor expansion, which implies that the tangential velocity at the edge of the EDL ($r\sim 1 + \lambda_{0}$) can be written as $u_{\theta } + \lambda_{0} \frac{{\partial u_{\theta } }}{\partial r}\sim (\beta + \lambda_{0} )\frac{{\partial u_{\theta } }}{{\partial u_{\theta } }}$. Finally, replacing (thin EDL) $\partial u_{\theta } /\partial r$ by $- u_{\theta } /\lambda_{0}$, we readily recover the same $1 + \beta /\lambda_{0}$ relation.

Note that under a no-slip condition ($\beta = 0$), Eq. (29) renders the traditional HS velocity slip relation [15], while the general HS velocity slip expression for a porous medium and slipping surface is provided by Eq. (28). The resistance parameter $R^{T} \left( {\alpha ,\beta } \right)$ given in Eq. (17) and the ICEP parameter $p(\alpha ,\beta ,\lambda_{0} )$ defined in Eq. (26) can both be analytically determined by using Eqs. (70), (77) and (78). Finally, it is worth noting that the general expression for the HS slip in Eq. (28) can be also extended for unrestricted EDLs, by means of employing the Teubner volume integral formulation in Eq. (16) and the asymptotic procedure outlined in Eq. (18).

## The ICEP force and travelling-wave dipolophoresis

Our next task is to evaluate the ICEP mobility of an ideally polarizable spherical hydrophobic colloid suspended in hydrogel (see Eq. (25)) that is induced by an arbitrary (axisymmetric) non-uniform ambient electric field defined in Eq. (4). By taking advantage of the following relationship [51] involving Legendre polynomials

30 $$ \left( {2n + 1} \right)\left( {1 - \mu^{2} } \right)\frac{{{\text{d}}P_{n} \left( \mu \right)}}{{{\text{d}}\mu }} = n\left( {n + 1} \right)\left[ {P_{n - 1} \left( \mu \right) - P_{n + 1} \left( \mu \right)} \right], $$

one finds from Eq. (25), employing Eq. (6) and the orthogonality property of the Legendre functions that

31 $$ F_{{{\text{ICEP}}}}^{\left( 1 \right)} = - 4\pi p\left( {\alpha ,\beta } \right)Re\mathop \sum \limits_{n = 1}^{\infty } \frac{{\left( {n + 1} \right)A_{n}^{*} A_{n + 1} }}{{\left( {n + 2 + i\Omega } \right)\left( {n + 1 - i\Omega } \right)}}, $$

where according to Eq. (6) and assuming thin EDL, $\Omega = \omega a\lambda_{0} /D$ denotes the dimensionless RC frequency.

Combining the DEP force expression from Eq. (8) with the corresponding ICEP component given in Eq. (31) renders the following equation for the total colloid (DIP) mobility prevailing along the *x*_1_ axis;

32 $$ U_{1} R^{T} = - \left( {F_{{{\text{DEP}}}}^{\left( 1 \right)} + F_{{{\text{ICEP}}}}^{\left( 1 \right)} } \right) = 2\pi {\text{Re}}\mathop \sum \limits_{n = 1}^{\infty } \frac{{\left( {n + 1} \right)A_{n}^{*} A_{n + 1} }}{n + 1 - i\Omega }\left[ {n + i\Omega + \frac{{2p\left( {\alpha ,\beta } \right)}}{n + 2 + i\Omega }} \right]. $$

Note that $p(\alpha ,\beta )$ is negative, and thus in general DEP and ICEP act in opposite directions. It is also interesting to note that for any ‘single-mode’ non-uniform ambient field, say $\chi_{am} (r,\mu ) = A_{n} P_{n(} \mu )r^{n}$ (including a uniform field *n* = 1 as a special case), the particle mobility is always null! Thus, as previously demonstrated, finite mobility can result only from an interaction between two adjacent (neighbouring) modes of the non-homogeneous applied field. Furthermore, for the particular case of a ‘two-mode’ excitation, involving for example a combination of a uniform field (A_1_) together with a ‘constant-gradient’ component (*A*_2_), under a DC forcing $\left( {\Omega = 0} \right)$ in a Stokes medium $\left( {\alpha = 0} \right)$ and non-slipping colloid $\left( {\beta = 0} \right)$ corresponding to *n* = 1 in Eq. (32), one has $p(0,0,\lambda_{0} )$ $= - 3/2$ (see Eq. (26) and Appendix) and thus the resulting DIP mobility of a spherical colloid is identical to zero, in agreement with [19, 21, 22]. However, it is evident from Eq. (32) that this rather unexpected result (i.e. vanishing mobility for interaction between the first and second modes) does not hold in general either for AC forcing $\left( {\Omega \ne 0} \right)$, for a Brinkman medium $\left( {\alpha \ne 0} \right)$ or Navier slipping surface $\left( {\beta \ne 0} \right)$.

To illustrate the general methodology and comply with the definition (up to a constant) of the ambient field (i.e. vanishing at the origin), let us consider the prevalent AC case of an axisymmetric unidirectional travelling-wave (TW) electric potential which is expressed in cylindrical coordinates $\left( {x_{1} ,\rho } \right)$ with $\rho^{2} = x_{2}^{2} + x_{3}^{2}$ as [45, 46]

33 $$ \chi_{am} \left( {x_{1} ,\rho } \right) = [I_{0} \left( {k\rho } \right)/k]{\text{sin}}\left( {kx_{1} - \omega t - \varphi } \right) = - {\text{Re}}\{ i[I_{0} \left( {k\rho } \right)/k]e^{{i\left( {kx_{1} - \varphi } \right) - i\omega t}} \} . $$

where *I*_0_ denotes the modified Bessel function of zeroth-order, *k* is the dimensionless wave number (wavelength scaled concerning the radius *a*), $\omega$ is the forcing frequency and $\varphi$ represents an arbitrary phase angle. The same approach can be also applied for a TW propagating along a 2D infinitely long narrow channel of width ${\text{w}}$ providing the wavelength is smaller than $w$ [43]. In conjunction with the ‘weak-field’ assumption, we also assume that the wavelength of the TW forcing is large compared to the size of the colloid (Rayleigh hypothesis), implying that $k < 1$. Thus, the limit of $k = 0$ corresponds to a constant potential or vanishing field and $k \ll 1$ represents ‘weak’ non-uniformity [52] (long-wavelength approximation). The ambient field in Eq. (33) satisfies Laplace’s equation and represents a time-harmonic wave propagating along the *x*_1_ axis with a constant wave celerity $a\omega /k$. Since the applied wave signal preserves azimuthal symmetry, it can also be expressed in terms of spherical coordinates as $\chi_{am} \left( {r,\mu } \right) = {\text{Re}}\left[ {\mathop \sum \nolimits_{n = 1} A_{n} P_{n} \left( \mu \right)r^{n} e^{ - i\omega t} } \right]$, where $x_{1} = r\mu$ and $r^{2} = x_{1}^{2} + \rho^{2}$. Note that along the *x*_1_ axis $\left( {i.e.,\, \rho = 0,r = x_{1} ,\left| \mu \right| = 1} \right)$, one gets after expanding $\exp \left( {ikx_{1} } \right)$ near the origin in a Taylor series in *kx*_1_ (using $I_{0} \left( 0 \right) = P_{n} \left( 1 \right) = 1$), the following expression for the (complex) amplitudes $A_{n}$ of the equivalent Legendre representation for the TW (‘sinusoidal’) forcing in Eq. (33);

34 $$ A_{n} = - i\left( {\frac{{k^{n - 1} }}{n!}} \right)e^{{i\left( {n\pi /2 - \varphi } \right)}} . $$

We recall that in the DC limit $\left( {\omega = 0} \right)$, only the real part of Eq. (34) should be considered, whereas in the AC case, the coefficients *A*_n_ are taken to be complex.

Let us next examine the time-independent (DC) case of a ‘stationary’ $\left( {\omega = 0} \right)$ simple ‘sinusoidal’ wave forcing, where according to Eq. (33) $\chi_{am} \left( {x_{1} ,\rho } \right) = [I_{0} \left( {k\rho } \right)/k]{\text{sin}}\left( {kx_{1} - \varphi } \right)$. Since the ambient field is spatially non-uniform, the conducting colloid is subject to both DEP and ICEP steady forces acting in the *x*_1_ direction. For example, F_DEP_ can be found directly from Eq. (8) by substituting $\Omega_{n} = 0 \left( {\omega = 0} \right),$ which for real $A_{n}$ readily results in [22–24]

35 $$ F_{{{\text{DEP}}}}^{\left( 1 \right)} = - 4\pi \mathop \sum \limits_{n = 1}^{\infty } nA_{n} A_{n + 1} . $$

Note again the difference by a factor of 2 between the DC and AC cases corresponding to Eqs. (35) and (8) due to the time-averaging operation. Thus, substituting the real values of Eq. (34) into Eq. (35) leads to the following closed-form expression

36 $$ F_{{{\text{DEP}}}}^{\left( 1 \right)} \left( k \right) = - 2\pi \sin \left( 2 \right)\mathop \sum \limits_{n = 1}^{\infty } \frac{{\left( { - 1} \right)^{n} k^{2n + 1} }}{{\left( {n - 1} \right){!}\left( {n + 1} \right){!}}} = - \frac{{2\pi J_{2} \left( {2k} \right)}}{k}\sin \left( {2\varphi } \right), $$

where $J_{n}$ denotes the ordinary Bessel function of order *n* (see [68] & 8.402). It is rewarding to note here that there exists a simple analytic solution for the DEP force exerted on a spherical polarizable colloid which is subject to a non-uniform stationary harmonic wave-field (of unit amplitude) proportional to $\sin (kx_{1} - \varphi )$. It is also worth mentioning that as expected this DEP force vanishes in the limit of an infinitely long wavelength, i.e. $k \to 0$ (corresponding to a uniform ambient potential), and for $\varphi = n\pi /2$ due to the symmetry of the wave signal with respect to the origin.

In a similar manner, one can also explicitly find the corresponding ICEP force [22] exerted on a free colloid under the same stationary wave forcing $\left( {\omega = 0} \right)$, by using Eq. (31) which was obtained for a hydrophobic spherical colloid embedded in a Brinkman medium

37 $$ \begin{aligned} F_{ICEP}^{\left( 1 \right)} \left( k \right) & = - 8\pi p\left( {\alpha ,\beta ,\lambda_{0} } \right)\mathop \sum \limits_{n = 1}^{\infty } \frac{{\left( {A_{n} A_{n + 1} } \right)}}{n + 2} \\ & = 4\pi p\left( {\alpha ,\beta ,\lambda_{0} } \right)\sin \left( {2\varphi } \right)\mathop \sum \limits_{n = 1}^{\infty } \frac{{\left( { - 1} \right)^{n} k^{2n - 1} }}{{n{!}\left( {n + 2} \right){!}}} \\ & = \left( {\frac{4\pi }{k}} \right)p\left( {\alpha ,\beta ,\lambda_{0} } \right)\left[ {\frac{{J_{2} \left( {2k} \right)}}{{k^{2} }} - \frac{1}{2}} \right]\sin \left( {2\varphi } \right) \\ \end{aligned} $$

Again, Eq. (37) provides a newly analytic solution for the ICEP force acting on a free hydrophobic spherical colloid placed in hydrogel solution, which is forced by a stationary (DC) ‘sinusoidal’ wave signal. It should be emphasized that in a similar manner to DEP, the ICEP force also vanishes for a zero wave number and zero phase angle and that both DEP and ICEP generally act in opposite directions.

Finally, let us consider the corresponding time-dependent AC ($$\omega \ne 0$$) case (see Eq. (33)), involving a travelling wave of zero phase $$\left( {\varphi = 0} \right)$$ propagating along the *x*_1_ axis with a finite wave celerity $$a\omega /k$$. Substituting the complex amplitudes $$A_{n} $$ in Eq. (34) into the corresponding AC expressions for the DEP and ICEP loads exerted on the colloid given in Eqs. (8) and (31) and assuming thin EDL readily yields

38 $$ \begin{aligned} F_{{{\text{DEP}}}}^{\left( 1 \right)} & = - 2\pi {\text{Re}}\mathop \sum \limits_{n = 1}^{\infty } \frac{{\left( {n + 1} \right)\left( {n - i\Omega } \right)}}{n + 1 + i\Omega }A_{n}^{*} A_{n + 1} , \\ F_{{{\text{ICEP}}}} & = - 4\pi p\left( {\alpha ,\beta ,\lambda_{0} } \right){\text{ Re}}\mathop \sum \limits_{n = 1}^{\infty } \frac{{\left( {n + 1} \right)A_{n}^{*} A_{n + 1} }}{{\left( {n + 1 + i\Omega } \right)\left( {n + 2 - i\Omega } \right)}}, \\ \end{aligned} $$

where $\Omega = \omega a\lambda_{0} /D$ denotes the dimensionless RC frequency [17]. One can easily check that in the DC limit $\left( {\Omega = 0} \right),$ Eqs. (38) reduce to Eqs. (35) and (37), after adjusting for the factor of 2 between the two cases.

Next, substituting the above values of the complex amplitudes A_n_ and taking the real parts of Eq. (38) renders

39 $$ F_{{{\text{DEP}}}}^{\left( 1 \right)} \left( k \right) = - 2\pi \Omega \mathop \sum \limits_{n = 1}^{\infty } \frac{{\left( {2n + 1} \right)k^{2n - 1} }}{{\left( {n{!}} \right)^{2} \left[ {\left( {n + 1} \right)^{2} + \Omega^{2} } \right]}}, $$

and

40 $$\begin{aligned} F_{{{\text{ICEP}}}}^{(1)} (k) &= - 4\pi p(\alpha ,\beta ,\lambda_{0} )\Omega \\ &\quad\times\mathop \sum \limits_{n = 1}^{\infty } \frac{{k^{2n - 1} }}{{\left( {n{!}} \right)^{2} \left[ {\left( {n + 1} \right)^{2} + \Omega^{2} } \right]\left[ {\left( {n + 2} \right)^{2} + \Omega^{2} } \right]}}.\end{aligned} $$

The total travelling-wave dipolophoretic (TWDIP) force acting on a free hydrophobic colloid suspended in a gel solute is readily found by summing up Eqs. (39) and (40), implying that the TWDIP-induced colloid mobility $U_{1} \left( {k,\Omega ;\alpha ,\beta } \right)$, is explicitly given by the following wave number-dependent asymptotic expansion

41 $$\begin{aligned} U_{1} &= \frac{2\pi k \Omega }{{R^{T} \left( {\alpha ,\beta } \right)}}\mathop \sum \limits_{n = 0}^{\infty } \frac{{k^{2n} }}{{\left[ {\left( {n + 1} \right)!]^{2} } \right[\left( {n + 2)^{2} + \Omega^{2} } \right]}}\\ & \left[ {2n + 3 + \frac{{2p\left( {\alpha ,\beta ,\lambda_{0} } \right)}}{{(n + 3)^{2} + \Omega^{2} }}} \right] \end{aligned}$$

This novel expression suggests for example that the leading–order term in the long-wavelength limit $\left( {k \ll 1} \right)$ of the mobility for a ‘clean’ solute $\left( {\alpha = 0} \right)$ and no-slip colloid $\left( {\beta = 0} \right)$ is proportional to $k\Omega \left( {8 + \Omega^{2} } \right)/[\left( {4 + \Omega^{2)} \left( {9 + \Omega^{2} } \right)} \right]$ $+ O(k^{2} )$, indicating a maximum at $\Omega \sim 2.$ It should also be mentioned that under a simple unidirectional sinusoidal travelling-wave (TW) excitation, both DEP and ICEP reactions (as well as the resulting colloid mobility) display a dispersion behaviour of a Lorentzian type of compact support, which is characterized by a distinct maximum and vanishing values for both $\Omega \to 0$ and $\Omega \to \infty$. Unlike the corresponding DC values (see Eqs. (36) and (37)), the DIP mobility is finite for $\varphi = 0$ and it tends to zero as expected in the infinitely long-wavelength limit $\left( {k \to 0} \right)$ (see also [44–46]).

## Induced-charge electroosmotic velocity field

Here, we consider the case of a Navier slipping spherical colloid embedded in a porous (Brinkman) medium which is subject to an arbitrary (axisymmetric)*‘single-mode’* non-uniform DC electric field given by $A_{n} r^{n} P_{n} \left( \mu \right),$ where $n$ is a positive integer representing for example the case of a uniform field (n = 1) or that of a constant-gradient (*n* = 2) [19, 45]. In the lack of any interaction between neighbouring (adjacent) modes, the resulting mobility of a spherical colloid is identical to zero. Nevertheless, due to polarization, there is an induced-charge electroosmotic (ICEO) flow field of symmetric pattern around the colloid [18]. In this sense, the incited flow field is similar in many respects to that around a rigid (no-slip) spherical particle freely suspended in a Stokes solute and driven by a uniform electric field [17, 18, 32, 69]. The resulting quadrupolar-type electroosmotic (EO) velocity field generally pumps liquid from infinity in the direction of the field and ejects fluid in the perpendicular direction [17, 31].

In order to determine the velocity field around a spherical colloid lying in a porous medium, let us first consider the following homogenous (unforced) dimensionless Brinkman equation (see Eq. (9)), where $\alpha$ denotes the Brinkman parameter (inverse of Darcy’s permeability) [31];

42 $$ \nabla P = \nabla^{2} {\varvec{u}} - \alpha^{2} {\varvec{u}}, \quad \quad \quad \nabla \cdot {\varvec{u}} = 0. $$

Equation (42) can also be written by virtue of the azimuthal symmetry (curl operation) in terms of the following differential operator [31, 34];

43 $$ E^{2} = \frac{{\partial^{2} }}{{\partial r^{2} }} + \frac{\sin \theta }{{r^{2} }}\frac{\partial }{\partial \theta }\left( {\frac{1}{\sin \theta }\frac{\partial }{\partial \theta }} \right); $$

thus, the radial and tangential components of the velocity field can be expressed in terms of the corresponding stream function $\Psi \left( {r,\theta } \right)$ of Eq. (42) governed by

44 $$ E^{2} \left( {E^{2} - \alpha^{2} } \right)\Psi \left( {r,\theta } \right) = 0, $$

where in the absence of a background stream, it is assumed that $\Psi \to 0$ as r $\to \infty$.

A general solution of the fourth-order partial differential Eq. (44) can be written as [55, 60];

45 $$ \Psi \left( {r,\theta } \right) = \mathop \sum \limits_{n = 2}^{\infty } \left[ {\tilde{B}_{n} r^{1 - n} + \tilde{C}_{n} \sqrt r K_{n - 1/2} \left( {\alpha r} \right)} \right]G_{n}^{{\left( { - 1/2} \right)}} \left( \theta \right), $$

where $K_{n - 1/2}$ denotes the spherical modified Bessel function of fractional order *n*^-1/2^, $G_{n}^{ - 1/2}$ represents the Gegenbauer polynomials of order *n* and degree -1/2 and $\left( {\tilde{B}_{n} ,\tilde{C}_{n} } \right)$ are coefficients to be determined. Imposing in Eq. (45), the boundary condition of an impermeable colloid (zero radial velocity) readily yields for *r* = 1;

46 $$ \tilde{B}_{n} = - \tilde{C}_{n} K_{n - 1/2} \left( \alpha \right). $$

The tangential component of the surface velocity ${\mathbf{u}}_{{\mathbf{s}}} (\theta ) = u_{\theta } (r = 1,\theta ){\mathbf{e}}_{{{\varvec{\uptheta}}}}$ along the hydrophobic spherical particle can be accordingly determined from Eq. (45) as

47 $$ \begin{aligned} &u_{\theta } \left( {1,\theta } \right)  = \frac{ - 1}{{r\sin \theta }}\frac{\partial \Psi }{{\partial r}}\left( {r,\theta } \right)|_{r = 1}  = \frac{ - 1}{{\sin \theta }}\mathop \sum \limits_{n = 2}^{\infty } \\ & \tilde{C}_{n} \left\{ {\frac{d}{dr}\left[ {\sqrt r K_{n - 1/2} \left( {\alpha r} \right)} \right]_{r = 1} + \left( {n - 1} \right)K_{n - 1/2} \left( \alpha \right)} \right\} \\ &  G_{n}^{( - 1/2)} \left( \theta \right)= \alpha \mathop \sum \limits_{n = 2}^{\infty } \left[ {\tilde{C}_{n} K_{n - 3/2} \left( \alpha \right)} \right]\frac{{G_{n}^{( - 1/2)} \left( \theta \right)}}{\sin \theta }. \\ \end{aligned} $$

The unknown coefficients $\tilde{C}_{n}$ can next be found by imposing the general slip velocity of a hydrophobic particle suspended in a Brinkman fluid given in Eq. (28).

Consider for example the case of a ‘single-mode’ electric forcing $\chi_{am} \left( {r,\mu } \right) = A_{M} r^{M} P_{M} \left( \mu \right)$ where M is any prescribed positive integer, and thus under Eqs. (4) and (6) (thin EDL), one gets

48 $$ \alpha \mathop \sum \limits_{n = 2}^{\infty } \tilde{C}_{n} K_{n - 3/2} \left( \alpha \right)\frac{{G_{n}^{{\left( { - 1/2} \right)}} \left( \mu \right)}}{{1 - \mu^{2} }} = 4w\left( {\alpha ,\beta ,\lambda_{0} } \right)\left| {A_{M} } \right|^{2} \frac{{\left( {2M + 1} \right)^{2} }}{{\left( {M + 1} \right)^{2} + \Omega^{2} }}P_{M} \left( \mu \right)\frac{{{\text{d}}P_{M} \left( \mu \right)}}{{{\text{d}}\mu }}. $$

To find $\tilde{C}_{n}$ from Eq. (48), we employ the following orthogonality relation for the Gegenbauer polynomials of order n and degree -1/2 (see [68] & 7.313.2);

49 $$ \mathop \int \limits_{ - 1}^{1} \frac{{G_{n}^{{\left( { - 1/2} \right)}} \left( \mu \right)G_{m}^{{\left( { - 1/2} \right)}} \left( \mu \right)}}{{1 - \mu^{2} }}{\text{d}}\mu = \frac{{\delta_{mn} }}{{n\left( {n - 1/2} \right)\left( {n - 1} \right)}},\quad n \ge 2, $$

where $\delta_{mn}$ denotes the Kronecker delta function which for A_M_ = 1 yields

50 $$\begin{aligned} &\alpha \tilde{C}_{n} \left( M \right)K_{n - 3/2} \left( \alpha \right) \\ & \quad = 4w\left( {\alpha ,\beta ,\lambda_{0} } \right)n\left( {n - 1/2} \right)\left( {n - 1} \right)\left| {A_{M} } \right|^{2}\\ & \quad  \mathop \int \limits_{ - 1}^{1} P_{M} \left( \mu \right)\frac{{dP_{M} \left( \mu \right)}}{d\mu }G_{n}^{{\left( { - 1/2} \right)}} \left( \mu \right){\text{d}}\mu .\end{aligned} $$

Note that both $G_{n}^{{\left( { - 1/2} \right)}} \left( \mu \right)$ and $P_{n} \left( \mu \right)$ are either even or odd polynomials of $\mu$ depending on whether *n* is even or odd. Thus, since the product $P_{M} \left( \mu \right)dP_{M} \left( \mu \right)/{\text{d}}\mu$ is always an odd polynomial of $\mu$, *n* must be an odd integer for the integral on the right-hand side of Eq. (50) to be finite. Hence, only odd orders (*n* = 2* m* + 1) of the Gegenbauer polynomials should be considered in Eq. (45). In addition, it follows from Eq. (48) that *m* and *M* should be both either even or odd.

The integral in Eq. (50) can be further simplified using integration by parts and noting that ${\text{d}}G_{n}^{{\left( { - 1/2} \right)}} \left( \mu \right)/{\text{d}}\mu = - P_{n - 1} \left( \mu \right)$ and $G_{2m + 1}^{{\left( { - 1/2} \right)}} \left( { \pm 1} \right) = 0$ for $m \ge 1$ (see [68] & 8.935.2 and 8.9.3.6.2), resulting in

51 $$ \mathop \int \limits_{ - 1}^{1} P_{M} \left( \mu \right)\frac{{{\text{d}}P_{M} \left( \mu \right)}}{{{\text{d}}\mu }}G_{2m + 1}^{{\left( { - 1/2} \right)}} \left( \mu \right){\text{d}}\mu = \frac{1}{2}\mathop \int \limits_{ - 1}^{1} \left[ {P_{M} \left( \mu \right)} \right]^{2} P_{2m} \left( \mu \right){\text{d}}\mu , $$

The integral on the right-hand side of Eq. (51) can then be evaluated using Neumann’s expansion for the product of two Legendre polynomials (see [51] p. 87). Thus, substituting Eq. (51) in Eq. (50) for $n = 2m + 1, m \ge 1$ renders

52 $$ \alpha \tilde{C}_{2m + 1} \left( M \right)K_{2m - 1/2} \left( \alpha \right) = 4w\left( {\alpha ,\beta ,\lambda_{0} } \right)\frac{{\left( {2M + 1} \right)^{2} }}{{\left( {M + 1} \right)^{2} + \Omega^{2} }}T\left( {m,M} \right), $$

where

53 $$ T\left( {m,M} \right) = m\left( {2m + 1/2} \right)\left( {2m + 1} \right)[A_{M} ]^{2} \mathop \int \limits_{ - 1}^{1} P_{2m} \left( \mu \right)[P_{M} \left( \mu \right)]^{2} {\text{d}}\mu $$

and the slip parameter $w\left( {\alpha ,\beta ,\lambda_{0} } \right)$ is defined in Eq. (28).

The final expression for the ICEO-induced stream function around a hydrophobic spherical colloid in a Brinkman medium under a non-uniform electric forcing $A_{M} r^{M} P_{M} (\mu )$ can then be found by substituting Eqs. (46), (52), and (53) in Eq. (45) resulting in the new expression

54 $$\begin{aligned} &\Psi (r,\theta ) = \frac{{4w(\alpha ,\beta ,\lambda_{0} )}}{\alpha }\frac{{(2M + 1)^{2} }}{{(M + 1)^{2} + \Omega^{2} }}\\ & \quad \sum\limits_{m = 1}^{M} {T(m,M)\frac{{K_{2m + 1/2} (\alpha )}}{{K_{2m - 1/2} (\alpha )}}} \\ & \quad \left[ {\sqrt r \frac{{K_{2m + 1/2} \left( {\alpha r} \right)}}{{K_{2m + 1/2} \left( \alpha \right)}} - \left( \frac{1}{r} \right)^{2m} } \right]G_{2m + 1}^{{\left( { - 1/2} \right)}} \left( \theta \right).\end{aligned} $$

The limit of Eq. (54) for a ‘clean’ non-porous Stokesian medium $\left( {\alpha = 0} \right)$ seems to be non-trivial.

However, by using the following expression for the modified Bessel function (see [68] & 8.468)

55 $$ K_{n + 1/2} \left( x \right) = \sqrt {\frac{\pi x}{2}} \frac{{e^{ - x} }}{{x^{n + 1} }} \mathop \sum \limits_{k = 0}^{n} \frac{{\left( {n + k} \right){!}x^{n - k} }}{{2^{k} k{!}\left( {n - k} \right){!}}} $$

one gets

56 $$ \mathop {{\text{lim}}}\limits_{\alpha \to 0} \left( {\sqrt r \frac{{K_{2m + 1/2} \left( {\alpha r} \right)}}{{K_{2m + 1/2} \left( \alpha \right)}} - \frac{1}{{r^{2m} }}} \right) \to \frac{{\alpha^{2} \left( {1 - r^{2} } \right)}}{{2\left( {4m - 1} \right)r^{2m} }} + O\left( {\alpha^{3} } \right) $$

and

57 $$ \mathop {{\text{lim}}}\limits_{\alpha \to 0} \frac{{\alpha K_{2m + 1/2} \left( \alpha \right)}}{{K_{2m - 1/2} \left( \alpha \right)}} \to \left( {4m - 1} \right) + O\left( \alpha \right) $$

Thus, one finds from Eq. (54) that for $\alpha = 0$ $\left( {r \ge 1} \right)$;

58 $$\begin{aligned}& \Psi (r,\theta ) = 2w(0,\beta ,\lambda_{0} )\\ &\frac{{\left( {2M + 1} \right)^{2} }}{{\left( {M + 1} \right)^{2} + \Omega^{2} }}\mathop \sum \limits_{m = 1}^{M} T\left( {m,M} \right)\frac{{\left( {1 - r^{2} } \right)}}{{r^{2m} }}G_{2m + 1}^{{\left( { - 1/2} \right)}} \left( \theta \right),\end{aligned} $$

which for example for the particular case of *M* = 1 and $A_{M} = 1$, namely for a *uniform* field excitation corresponding to $\chi_{am} \left( {r,\theta } \right) = - rcos\theta = - x_{1}$, Eq. (58) simply reduces to

59 $$ \Psi (r,\theta ) = - \frac{{18w(0,\beta ,\lambda_{0} )}}{{4 + \Omega^{2} }}\left( {1 - \frac{1}{{r^{2} }}} \right)\sin^{2} \theta \cos \theta $$

since $G_{3}^{{\left( { - 1/2} \right)}} \left( \theta \right) = \left( {\sin^{2} \theta \cos \theta } \right)/2$ and following Eq. (53), one gets *T* (1, 1) = 2. Furthermore, for a ‘clean’ non-porous medium $(\alpha = 0)$ and a non-slip colloid $\left( {\beta = 0} \right)$, we have (see &. 4) p = -3/2, $R^{T} = - 6\pi$, $w = 1/4$ and thus Eq. (59) renders

60 $$ \Psi \left( {r,\theta } \right) = - \frac{9}{{2\left( {4 + \Omega^{2} } \right)}}\left( {1 - \frac{1}{{r^{2} }}} \right)\sin^{2} \theta \cos \theta $$

in full agreement with the known quadrupolar solution given for example in [17]. The corresponding tangential and radial components of the ICEO velocity field prevailing in the hydrogel medium can then be readily found from the explicit expression of the Stokes stream function given in Eq. (54).

Finally, we consider the intricate DC case of a simple ‘sinusoidal’ forcing, namely when the ambient field along the axis of symmetry (*x*_1_) is proportional to $\sin (kx_{1} )/k$, so that as $k \to 0$ (long-wavelength limit) the field is uniform. The corresponding Legendre coefficients (real) of this wave form following Eq. (34) are all-odd and are given by $A_{2n + 1} = \left( { - 1} \right)^{n} k^{2n} /\left( {2n + 1} \right){!}$. The induced slip velocity on the surface of the colloid can be thus expressed according to Eqs. (4) and (28) as

61 $$\begin{aligned} &u_{\theta } \left( {1,\theta } \right) = \frac{{w\left( {\alpha ,\beta ,\lambda_{0} } \right){\text{sin}}\theta }}{2}\frac{\partial }{\partial \mu }\\ & \quad \mathop \sum \limits_{l = 0} \mathop \sum \limits_{n = 0} \frac{{\left( {4l + 3} \right)\left( {4n + 3} \right)\left( { - 1} \right)^{l + n} }}{{\left( {l + 1} \right)\left( {n + 1} \right)\left( {2l + 1} \right){!}\left( {2n + 1} \right){!}}}\\ & \qquad k^{2l + 2n} P_{2l + 1} \left( \mu \right)P_{2n + 1} \left( \mu \right).\end{aligned} $$

It is clear following Eq. (61) that under this particular DC wave signal $\left( {{\Omega } = 0} \right),{ }$ ${\text{which}}\;{\text{is}}\;{\text{expressed}}\;{\text{only}}\;{\text{in}}\;{\text{terms}}\;{\text{of}}\;{\text{odd}}\;{\text{coefficients}}, {\text{ one}}\;{\text{has}}\;{ }A_{m} \cdot A_{m + 1} = 0$ and thus the colloid mobility is null! Nevertheless, the same ‘sine-wave’ forcing induces a symmetric electroosmotic (EO) flow field around the freely suspended hydrophobic particle in a gel, where the corresponding Stokes stream function is given by (see Eqs. (45) and (46))

62 $$\begin{aligned} \Psi \left( {r,\theta } \right) &= \mathop \sum \limits_{m = 1} \tilde{C}_{2m + 1} K_{2m + 1/2} \left( \alpha \right)\\ & \quad \left[ {\sqrt r \frac{{K_{2m + 1/2} \left( {\alpha r} \right)}}{{K_{2m + 1/2} \left( \alpha \right)}} - \left( \frac{1}{r} \right)^{2m} } \right]G_{2m + 1}^{{\left( { - 1/2} \right)}} \left( \theta \right).\end{aligned} $$

The unknown coefficients $\tilde{C}_{2m + 1}$ in Eq. (62) can be accordingly found by employing Eq. (46) together with the orthogonality properties of the Gegenbauer polynomials in Eq. (49), which renders for $m \ge 1$;

63 $$\begin{aligned} \tilde{C}_{2m + 1} &= \frac{{m\left( {2m + 1} \right)\left( {4m + 1} \right)w\left( {\alpha ,\beta ,\lambda_{0} } \right)}}{{\alpha K_{2m - 1} \left( \alpha \right)}}\mathop \sum \limits_{l = 0} \mathop \sum \limits_{n = 0} \\ & \frac{{\left( {4l + 3} \right)\left( {4n + 3} \right)\left( { - 1} \right)^{l + n} }}{{\left( {l + 1} \right)\left( {n + 1} \right)\left( {2l + 1} \right){!}\left( {2n + 1} \right){!}}}I\left( {l,n,m} \right)k^{2l + 2n} .\end{aligned} $$

where

64 $$ I\left( {l,n,m} \right) = \mathop \int \limits_{ - 1}^{1} P_{2l + 1} \left( \mu \right)P_{2n + 1} \left( \mu \right)P_{2m} \left( \mu \right){\text{d}}\mu . $$

The triple-Legendre polynomial in Eq. (64) can be explicitly expressed in terms of the Wigner coefficient function [70] and for the sake of completeness is given below as

65 $$ I\left( {l,n,m} \right) = \frac{2}{2m + 2n + 2l + 3}\frac{{\tau_{m + n - l} \tau_{m + l - n} \tau_{l + n - m + 1} }}{{\tau_{m + l + n + 1} }},\quad \quad \tau_{n} = \frac{{\left( {2n} \right){!}}}{{2^{n} \left( {n{!}} \right)^{2} }} $$

By letting $k \to 0$ (corresponding to a uniform ambient field) in Eq. (63), one finds that $l = n = 0, m = 1$ and following Eq. (64) $I\left( {0,0,1} \right) = 4/15$. Substituting these values in Eqs. (62) and (63), render the sought solution for the stream function of the ICEO velocity field engendered around a hydrophobic spherical polarizable colloid suspended in a Brinkman medium due to a uniform electric field (unit amplitude)

66 $$\begin{aligned} \Psi \left( {r,\theta } \right) &= \frac{{36w\left( {\alpha ,\beta ,\lambda_{0} } \right)K_{5/2} \left( \alpha \right)}}{{\alpha K_{3/2} \left( \alpha \right)}}\\ & \left[ {\sqrt r \frac{{K_{5/2} \left( {\alpha r} \right)}}{{K_{5/2} \left( \alpha \right)}} - \left( \frac{1}{r} \right)^{2} } \right]G_{3}^{{\left( { - 1/2} \right)}} \left( \theta \right),\end{aligned} $$

where the slip parameter $w(\alpha ,\beta ,\lambda_{0} )$ is defined in Eq. (28). It can be easily verified that for a free solution $(\alpha \to 0)$, Eq. (66) reduces as expected to the DC limit $(\Omega \to 0)$ of Eq. (60).

Recalling next that the azimuthal vorticity component induced in the gel can be expressed in terms of the Stokes stream function as [34] $\varpi_{\varphi } = - E^{2} \Psi \left( {r,\theta } \right)/\left( {r\sin \theta } \right)$ and thus one finds from Eq. (66) that

67 $$ \varpi_{\varphi } \left( {r,\theta } \right) = - 9w\left( {\alpha ,\beta ,\lambda_{0} } \right)\frac{{K_{5/2} (\alpha r)}}{{\sqrt r \;K_{3/2} (\alpha )}}\sin (2\theta ), $$

exhibiting an exponential radial decay like $\frac{{e^{{ - \alpha \left( {r - 1} \right)}} \left( {\frac{{\alpha^{2} }}{r} + \frac{3\alpha }{{r^{2} }} + \frac{3}{{r^{3} }}} \right)}}{{\left( {1 + \alpha } \right)}}$ and implying that the vorticity at the colloid surface increases with the Darcy coefficient $\alpha$. It is noteworthy that for a free solute $\left( {\alpha = 0} \right),$ we obtain again the proper $1/r^{3}$ vorticity decay as in regular quadrupolar flows [17]. Using the same methodology, one can also determine the following dimensionless expression for the tangential shear stress along the hydrophobic colloid $(r = 1)$:

68 $$ \tau_{r\theta } \left( {1,\theta } \right) = 9w\left( {\alpha ,\beta ,\lambda_{0} } \right)\left[ {2 + \frac{{\alpha^{2} + 3\alpha + 3}}{\alpha + 1}} \right]\sin (2\theta ), $$

indicating that there is no flow separation for any value of $\alpha$ and the existence of four stagnation points on the colloid surface. The surface skin friction increases with the Darcy parameter and for a free solution $(\alpha = 0)$, the square parenthesis in Eq. (68) is equal as expected to 5 due to the $1/r^{4}$ radial dependence of the tangential velocity [17] (see Eq. (60)).

**Fig. 1:**
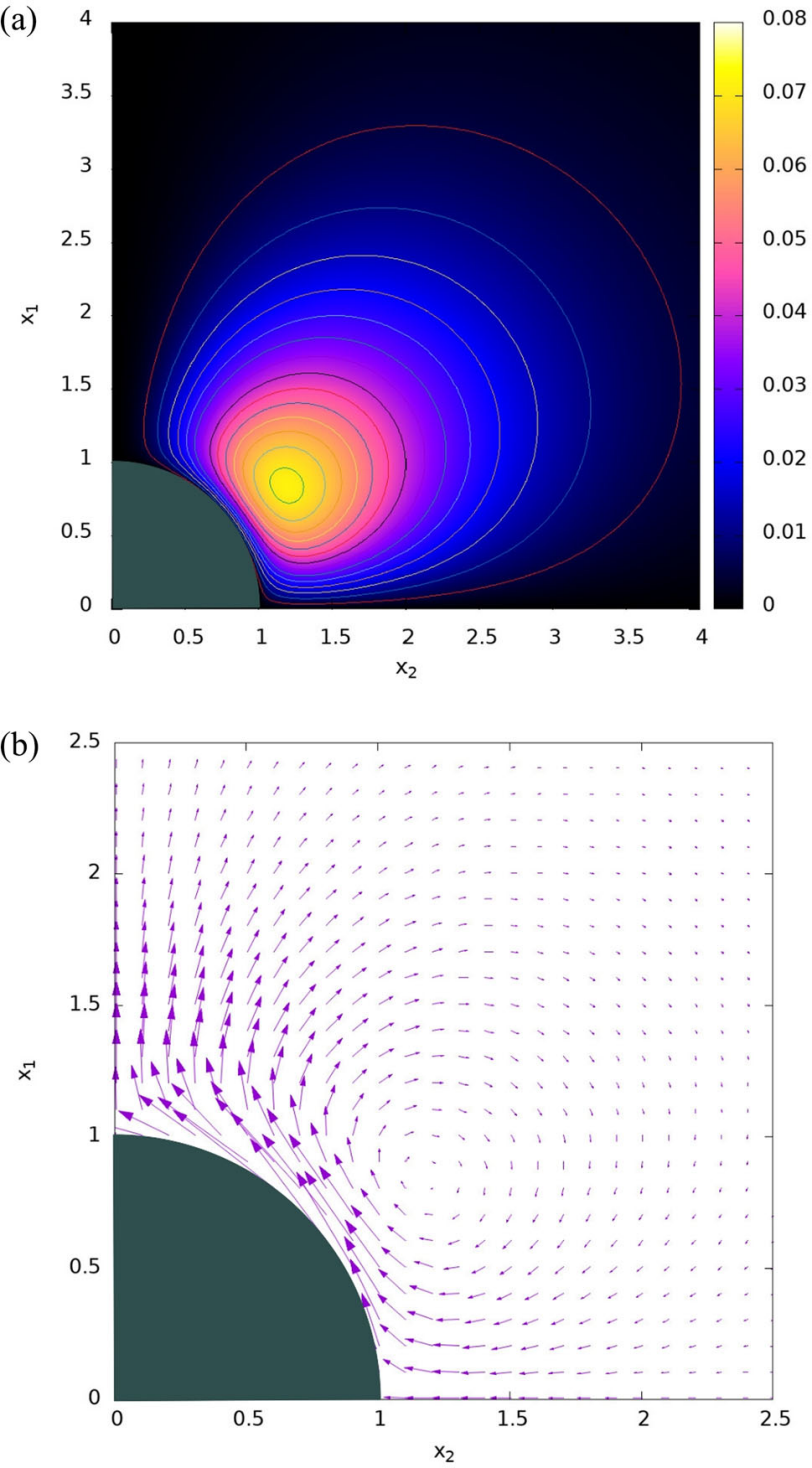
Contours of the **a** stream function and **b** corresponding velocity vector following Eq. (66) and for $\alpha = 1$, where $w\left( {\alpha ,\beta ,\lambda_{0} } \right)$ was taken as 1/72 for the contour levels plotted in **a**. Only one quarter of the flow field plane is plotted as the particle is stationary in the short wave limit and hence other quarters are mirror images

For finite values of the Brinkman coefficient $\alpha$, the first (short-range) term in the square parenthesis of Eq. (66) decays exponentially away from the surface and the second (long-range) term corresponds to the potential quadruple [34]. Note that Eq. (66) which was obtained as the long-wavelength limit of a sinusoidal wave form also applies for the case of a uniform forcing depending on the Darcy ($\alpha$) and Navier ($\beta$) parameters. For a free $\left( {\alpha = 0} \right)$ solute, one recovers given Eqs. (56) and (57), the DC $\left( {\Omega = 0} \right)$ limit of the corresponding AC expression in Eq. (59) (multiplied by 2), which for a non-slip case $\left( {\beta = 0} \right)$ reduces to the quadrupolar ICEO streamline pattern given in [33]. Typical plots of the stream function (Eq. 66) and the associated velocity field of a free uncharged spherical colloid lying in a hydrogel solution are given in Fig. 1 for $\alpha = 1$. The colloid is stationary as the excitation’s wave number *k* = 0 (see Eq. (41) for the colloid’s dimensionless DIP mobility expression). The fluid has its maximum velocity on the colloid’s surface at about $\theta = 45^{0}$, demonstrating a structure of a vortex ring around that latitude. Increasing $\alpha$ will result in reducing the flow penetration into the hydrogel due to a decrease in the gel’s permeability.

**Fig. 2:**
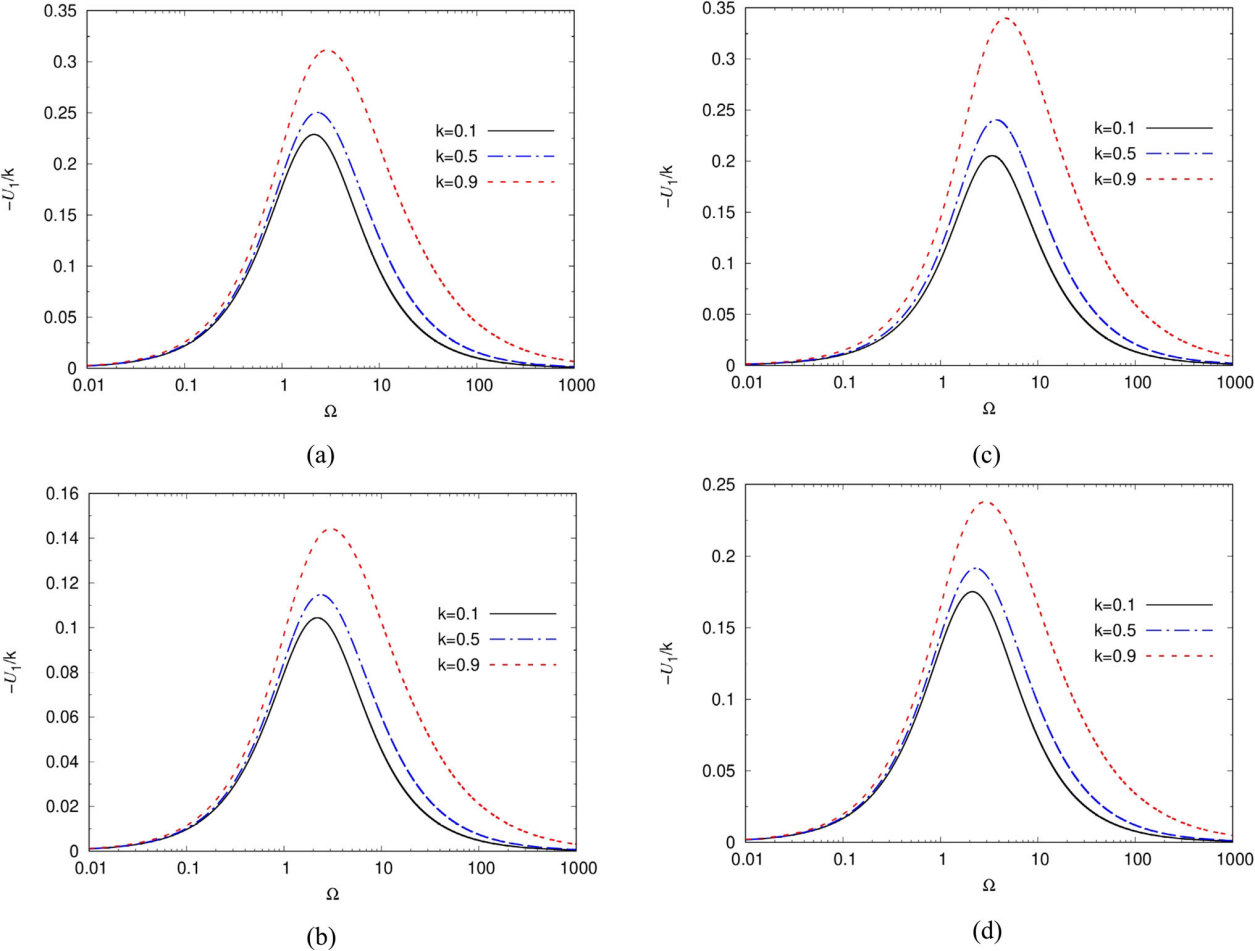
Variation of the colloid’s dimensionless mobility $U_{1}$ with the excitation’s dimensionless RC frequency ${\Omega }$ for three dimensionless wave numbers k’s following Eq. (41), and **a** $\left( {\alpha ,\beta } \right) = \left( {0,0} \right)$, **b** $\left( {\alpha ,\beta } \right) = \left( {1,0} \right)$, **c** $\left( {\alpha ,\beta } \right) = \left( {0,1} \right)$, and **d** $\left( {\alpha ,\beta } \right) = \left( {1,1} \right)$, where $\lambda_{0} = 1$

The variation of the colloid’s dimensionless mobility *U*_1_ with the excitation dimensionless frequency ${\Omega }$ is given in Fig. 2 for several dimensionless wave numbers *k* and $0 \leqq \alpha , \beta  \leqq 1$ using the newly derived Eq. (66). The mobility *U*_1_ is divided by *k* in Fig. 2 to illustrate the dominance of the first mode (*m* = 0) in the series solution of Eq. (41), particularly for small values of *k*, which result in a small difference between the DIP mobility at *k* = 0.1 and 0.5 when looking at *U*_1_/k. As *k* increases, obviously *U*_1_ increases, but also its maximum shifts towards higher frequencies due to the effect of higher terms in the series of Eq. (41). Nevertheless, one may conclude that by considering only the first terms (*m* = 0) in Eq. (41), it is still possible to get a reasonably accurate solution for *k* < 0.5 as illustrated in Fig. 2. Increasing *k* above 1 results in rather slow convergence of the series in Eq. (41) and thus the figure is limited to *k* < 1 (Rayleigh’s approximation). This is of high relevance for when this model is used as a sub-grid model to simulate the motion of multi-colloids (assuming two ways interaction).

Increasing the Brinkman friction coefficient $\alpha$ from 0 to 1 reduces the DIP mobility due to a decrease in the gel’s permeability, but has otherwise a minimal effect on the locations of the maxima. On the other hand, increasing the Navier slip coefficient $\beta$ from 0 to 1 tends to enhance the colloid’s mobility as the velocity condition on the surface furthers away from the non-slip one, while also shifting the maxima’s locations to higher wave numbers. Such finding is of importance for the design of colloids systems. Having found the ICEO velocity of a single particle, one can also determine, for example, the long-range interaction force between two equal-size remote (large–spacing) ideally polarizable particles suspended in hydrogel under a uniform ambient field, using the methodologies of [25, 71, 72] and Faxen’s law [34, 64].

## Discussion and summary

A general unified framework is presented for solving the nonlinear DIP mobility problem of hydrophobic ideally polarized uncharged spherical colloids freely suspended in a hydrogel solute under arbitrary DC/AC non-uniform electric fields. The analytic solutions thus obtained depend on two parameters (length scales): the Brinkman-Debye-Bueche coefficient $\alpha$ characterizing the porosity of the porous liquid medium and the Navier–Maxwell–Basset coefficient $\beta$ relating to the velocity slip on the surface of the hydrophobic particle. In the limiting case of a free electrolyte solution ($\alpha = 0$) and non-slipping surface $\left( {\beta = 0} \right),$ the newly found solutions are compared against available expressions reported in the literature. In order to make the analysis amenable, we made the following assumptions: the amplitude of the ambient field is considered small compared to the thermal—scale (‘weak field’), and the inertia term can be neglected with respect to the corresponding electrostatic and diffusion terms (small Peclet). In addition, we take the binary electrolyte to be $z - z$ symmetric with equal diffusivities. Thus, the nonlinear Poisson–Nernst–Planck system of equations can be solved by means of linearization due to the uncoupling between the electrostatic and the hydrodynamic problems.

Next, we consider an arbitrary non-uniform steady (DC) or time-dependent (AC) ambient field represented by a series of spherical harmonics which is expressed in terms of Legendre polynomials (see Eq. (4b)) and apply the method of multipoles to determine the dielectrophoretic (DEP) force acting on the free colloid in terms of the prescribed amplitudes of the applied field and the RC frequencies. As a result of the interaction between the charge distribution in the electrolyte and the ambient field, the resulting electroosmotic (EO) velocity field exerts an additional hydrodynamic induced-charge electrophoretic (ICEP) force, which can be explicitly found utilizing Teubner’s integral formulation for unrestricted electric double layers (EDL). The total mobility of a freely suspended particle is thus determined by the combination of DEP + ICEP, which is often referred to as dipolophoresis (DIP). It is also worth mentioning that in general, DEP and ICEP tend to act in opposite directions and that ICEP theoretical predictions are usually over-estimated compared to experimental results (see discussions in [73–75]).

Experimental observations indicate that the theoretical value of the HS slip velocities (thin EDL) are generally smaller compared to the measured values and thus affecting the theoretical predictions of ICEP and ICEO. For this reason, a constant “correction factor” $\Lambda \le 1,$ is often used to get a better matching between theory and measurements. The theoretical value is clearly $\Lambda = 1$ but in practice, this empirical factor can be smaller by one order of magnitude compared to unity (see Table 1 in [74]). The physical reasons for the reduction in the measured values of the slip velocities compared to the theoretical predictions can be attributed to various physical effects, such as dielectric coating, Stern layer capacitance, surface roughness, ion absorption and counterion crowding, flow instability and Faradaic reactions [75]. In order to incorporate the above correction factor in our analysis, one can simply replace in Eqs. (28, 32, 38, and 41) the parameter $p\left( {\alpha ,\beta ,\lambda_{0} } \right)$ defined in Eq. (26) by $\Lambda p(\alpha ,\beta$, which accordingly reduces the ICEP contribution against DEP in the analytic expressions for the DIP mobility.

The final expression for the particle DIP mobility ${\varvec{U}}$ is obtained by using the Lorentz reciprocal theorem and is presented as a volume integral in Eq. (16) in terms of the solution for the auxiliary velocity ${\varvec{u}}^{{\left( {\varvec{i}} \right)}}$ governed by Eqs. (12) and (13), and the resistance coefficient $R^{T} \left( {\alpha ,\beta } \right)$ defined in Eq. (17). Further simplifications are possible under the common assumption of a thin ($\lambda_{0} \ll 1$) EDL. This renders asymptotic expansions involving surface (instead of volume) integrals and an additional modified slip parameter $p(\alpha ,\beta ,\lambda_{0} )$ defined in Eq. (26), which is determined by the solution of the auxiliary problem. The leading order in $\lambda_{0}$ ICEP expansion for perfectly conducting hydrophobic colloids is given in Eq. (25). Also presented in Eq. (28) is an asymptotic solution for the modified HS slip velocity depending on the Brinkman parameter $\alpha$, the Navier slip $\beta$ and (small) EDL thickness $\lambda_{0}$, which is subsequently used to determine the ICEO flow field engendered around a stationary colloid. In this case, the solution reduces to the corresponding expression for the HS velocity presented in [37–39] for a free Stokesian solute ($\alpha = 0$) and to the classical HS slip relation under no-slip condition ($\beta = 0$).

The general DIP solutions thus found for an arbitrary non-uniform AC electric forcing are next demonstrated for the particular case of a travelling-wave (TW) ‘sinusoidal’ excitation in the form of $$k^{ - 1} \sin (kx - \omega t - \varphi )$$, with prescribed wavelength ($$k$$), frequency ($$\omega$$) and arbitrary phase ($$\varphi$$). The DC limit ($$\omega = 0$$) is first discussed and new analytical solutions for both DEP and ICEP forces are given respectively in Eqs. (36) and (37) depending on $$\left( {k,\varphi } \right).$$ It is shown as expected that these two force components vanish due to symmetry for $$\varphi = 0$$ and for an infinitely long wave ($$k \to 0$$), which corresponds to a uniform field. Also presented in Eqs. (39) and (40) is a new explicit AC travelling-wave solution for $$\varphi = 0$$, expressed in terms of the dimensionless RC frequency ($$\Omega = \omega a\lambda_{0} /D$$), the ambient field wave number $$k$$ and the problem parameters $$(\alpha ,\beta ,\lambda_{0} )$$$$.$$ We show that both DEP and ICEP vanish as expected for $$\Omega \to 0$$, in accordance with the previously found DC solution. The DIP force and the resulting particle mobility exhibit a dispersion behaviour of a Lorentzian type where the DIP motility tends to zero both for infinitely small (DC) and large frequencies, displaying distinct maxima at moderate frequencies of $$O\left( 1 \right) $$(see for example Fig. 2). Once the DIP force is found, the colloid mobility can be readily obtained as in Eq. (41) in terms of the resistance (to translation) parameter $$R^{T} \left( {\alpha ,\beta } \right)$$ given in Eq. (17).

Next, we consider the intricate case of a spatially ‘symmetric’ non-uniform electric forcing resulting in zero mobility and analyse the induced-charge electroosmotic (ICEO) flow field generated around a polarized hydrophobic colloid placed in a liquid gel solution. Towards this goal, we examine the homogeneous (unforced) Stokes-Brinkman Eq. (41) which is driven by the surface slip velocity provided by Eq. (28). A convenient way of determining the resulting axisymmetric flow field is by employing a Stokes stream function formulation (Eq. (45)) for a stationary rigid slipping particle. First, we discuss the AC case of a ‘single-mode’ excitation, in which the phasor of the non-uniform ambient field is represented by a single harmonic $A_{M} r^{M} P_{M} \left( \mu \right),$ where $M$ is an arbitrary positive integer. The explicit solution for the stream function is then presented in Eq. (54) in terms of the relevant problem parameters $\left( {\alpha ,\beta ,\Omega ,M} \right).$ Some limiting cases of the above solution can also be readily obtained. For example, for a ‘clean’ (non-porous) electrolyte ($\alpha = 0$), one gets Eq. (59), which further reduces for $M = 1$ (uniform field) to the well-known quadrupolar ICEO solution [17] given in Eq. (60).

Finally, we examine the intriguing case where the free colloid is exposed to a simple (spatially inhomogeneous) ‘sinusoidal’ wave form given by $k^{ - 1} \sin (kx_{1} )$, which renders a uniform field with respect to *x*_1_ as $k \to 0.$ Under such a DC forcing, it is evident that the colloid mobility is null since all coefficients $(A_{n} )$ of the applied field are odd (see for example Eq. (32)), yet a symmetric ICEO flow field (resulting in fluid mixing and pumping) is generated around the particle with a wave number-dependent stream function given in Eq. (62). The general solution for the stream function in a Brinkman fluid under a non-uniform forcing is given in terms of spherical Bessel functions of the second kind and the Gegenbauer polynomials. This solution can be further reduced in the limit of $\alpha \to 0$(see Eq. (58)) and compared against the existing solution. In the long-wave limit, which corresponds for example to a uniform applied field, we recover the well-known quadrupolar solution for a non-porous medium and no-slip spherical colloid (see Eq. (60)). In addition to the ICEO velocity field incited in a hydrogel, we also provide analytic solutions for the azimuthal vorticity generated in the liquid gel and the variation of the skin friction coefficient along the surface of the hydrophobic rigid colloid.

## Conclusion

This work aimed to enhance our understanding of the dynamics (motility) of freely suspended (non-interacting) conducting hydrophobic colloids in hydrogel under arbitrary AC/DC non-uniform electric fields, and especially under the common case of a ‘sinusoidal’ travelling-wave excitation [50]. New explicit expressions were given for the colloid’s mobility (Eq. (41)) pointing to the mobility’s dependence on the excitation frequency and wave number and the occurrence of a maximum. In addition, we analytically explored the induced-charge electroosmotic (ICEO) velocity field around the particle including the distribution of the azimuthal vorticity and skin friction over the colloid’s surface (Eqs. (67, 68)), demonstrating how the resultant propelling force occurs which may affect future colloid designs. It is expected that the newly theoretical results thus presented will be compared in the future against experimental data involving slipping (hydrophobic) spherical colloids embedded in a porous hydrogel conducting fluid media. Nevertheless, we already see the derived theory as Eq. (41), a useful sub-grid model for the simulations of a sparse multi-colloid system using a two-way interaction, i.e. between a colloid and fluid.

## Data Availability

Data sets generated during the current study are available from the corresponding author on reasonable request.

## Appendix: Auxiliary hydrodynamic problem

Here, we consider the fundamental hydrodynamic problem of a spherical particle translating steadily with a uniform velocity *U*_i_ in an unforced (homogeneous) Stokes-Brinkman fluid medium that is subject to a (constant) Navier slip. Following [34, 76, 77], employing the method of Stokes’ singularities and using the analogy between the unsteady Stokes and Brinkman equations for incompressible flows, the velocity field generated in the (unbounded) fluid due to a moving rigid sphere can be expressed in term of the free-space Green function (oscillating Stokeslet) [34] as;

69 $$ S_{ij} \left( {\varvec{x}} \right) = \frac{{\delta_{ij} }}{r}A\left( {\alpha r} \right) + \frac{{x_{i} x_{j} }}{{r^{3} }}B\left( {\alpha r} \right), $$

where $A\left( z \right) = 2e^{ - z} \left( {1 + 1/z + 1/z^{2} } \right) - 2/z^{2}$ and $B\left( z \right) = - 2e^{ - z} \left( {1 + 3/z + 3/z^{2} } \right) + 6/z^{2}$. Note also that as expected $A\left( 0 \right) = B\left( 0 \right) = 1$.

Next, using the Faxen theorem and the relation [34]

70 $$ D_{ij} \left( {\varvec{x}} \right) = \frac{{ - \delta_{ij} }}{{r^{3} }}\delta_{ij} C\left( {\alpha r} \right) + \frac{{3x_{i} x_{j} }}{{r^{5} }}H\left( {\alpha r} \right), $$

where $D_{ij}$ represents the potential dipole term, $C\left( z \right) = e^{ - z} \left( {1 + z + z^{2} } \right)$ and $H\left( z \right) = e^{ - z} \left( {1 + z + z^{2} /3} \right)$. The velocity field engendered around the sphere of unit radius can be thus written as

71 $$ \hat{u}_{j}^{\left( i \right)} \left( {\hat{\user2{x}}} \right) = S_{ij} \left( {\hat{\user2{x}}} \right)b\left( {\alpha ,\beta } \right) + D_{ij} \left( {\hat{x}} \right)d\left( {\alpha ,\beta } \right), $$

where $b\left( {\alpha ,\beta } \right)$ and $d\left( {\alpha ,\beta } \right)$ are two coefficients to be determined and $\hat{u}_{j}^{(i)}$ represents the *j*^th^ component of $\hat{u}^{{\left( {\varvec{i}} \right)}} ,$ which denotes the velocity field of a steadily moving spherical colloid along the axis *x*_*i*_ [32, 34, 64]. Imposing the non-penetration boundary conditions (zero normal velocity) on *r* = 1 and using Eq. (71) leads to

72 $$ \left[ {A\left( \alpha \right) + B\left( \alpha \right)} \right]b\left( {\alpha ,\beta } \right) + \left[ { - C\left( \alpha \right) + 3H\left( \alpha \right)} \right]d\left( {\alpha ,\beta } \right) = 1, $$

In order to implement the velocity Navier slip boundary condition stated in Eq. (10), namely

73 $$ \left( {\hat{u}_{k}^{\left( i \right)} - \delta_{ik} } \right)\left( {\delta_{jk} - n_{j} n_{k} } \right) = \beta \left( {\hat{\sigma }_{km}^{\left( 1 \right)} n_{m} } \right)\left( {\delta_{jk} - n_{j} n_{k} } \right)\quad {\text{on}}\quad { }r = 1, $$

where $$n_{i} = x_{i} /a $$, we recall that following Eq. (71), the surface traction exerted on the sphere can be expressed in terms of the stress tensor $$\hat{\sigma }_{ij}^{\left( i \right)}$$ as;

74 $$ \hat{\sigma }_{jk}^{\left( i \right)} n_{k} = \delta_{ij} \left[ {b\left( {\alpha ,\beta } \right)K\left( \alpha \right) + d\left( {\alpha ,\beta } \right)M\left( \alpha \right)} \right] + n_{i} n_{j} \left[ {b\left( {\alpha ,\beta } \right)L\left( \alpha \right) + d\left( {\alpha ,\beta } \right)N\left( \alpha \right)} \right], $$

where [34]

75 $$ \begin{aligned} K\left( z \right) & = - 2\left[ {e^{ - z} \left( {z + 3 + \frac{6}{z} + \frac{6}{{z^{2} }}} \right) - \frac{6}{{z^{2} }}} \right], \\  L\left( z \right) &= 2\left[ {e^{ - z} \left( {z + 7 + \frac{18}{z} + \frac{18}{{z^{2} }}} \right) - \frac{18}{{z^{2} }} - 1} \right] \\ M\left( z \right) & = e^{ - z} \left( {6 + 6z + 3z^{2} + z^{3} } \right), \\ N\left( z \right) &= - e^{ - z} \left( {18 + 18z + 7z^{2} + z^{3} } \right), \\ \end{aligned} $$

Substituting Eqs. (69) to (71) together with Eq. (74) into Eq. (73) yields after some algebra (see also [35]), the following explicit expressions for the unknown coefficients $b\left( {\alpha ,\beta } \right)$ and $d\left( {\alpha ,\beta } \right)$ appearing in Eq. (71), i.e.

76 $$ \begin{aligned}b\left( {\alpha ,\beta } \right) &= \frac{3}{4}\left[ {\frac{{\left( {1 + \alpha } \right)\left( {1 + 2\beta } \right)}}{{1 + \beta \left( {3 + \alpha } \right)}} + \frac{{\alpha^{2} }}{3}} \right],\\ d\left( {\alpha ,\beta } \right) &= \frac{3}{{2\alpha^{2} }}\left[ {\frac{{\left( {1 + \alpha - e^{\alpha } } \right)\left( {1 + 2\beta } \right)}}{{1 + \beta \left( {3 + \alpha } \right)}} + \frac{{\alpha^{2} }}{3}} \right].\end{aligned} $$

One can then easily verify from Eq. (76) that for a non-porous medium and no-slipping surface, $b\left( {0,0} \right) = 3/4$ and $d\left( {0,0} \right) = - 1/4,$ which when substituted in Eq. (71) together with Eq. (69) (Stokeslet) and Eq. (70) (potential dipole) yields the classical solution in terms Stokes singularities for the velocity field of a translating no-slip rigid sphere [64].

Next, integrating the expression for the surface traction given in Eq. (74) over the particle surface yields the following expression for the dimensionless hydrodynamic drag exerted on the colloid:

77 $$\begin{aligned} \delta_{ij} R^{T} &= \mathop \int \limits_{S} \hat{\sigma }_{jk}^{\left( i \right)} n_{k} {\text{d}}s = 4\pi \delta_{ij} \Bigg\{ b\left( {\alpha ,\beta } \right)\left[ {K\left( \alpha \right) + \frac{1}{3}L\left( \alpha \right)} \right] \\ & \quad + d\left( {\alpha ,\beta } \right)\left[ {M\left( \alpha \right) + \frac{1}{3}N\left( \alpha \right)} \right] \Bigg\}, \end{aligned}$$

which by virtue of Eq. (76) results in the solution for $R^{T} \left( {\alpha ,\beta } \right)$ given in Eq. (17).

Finally, we need to evaluate (see Eq. (21)), the value of $\partial \hat{u}_{j}^{\left( i \right)} /\partial r$ at *r* = 1. Thus, following Eq. (71), one gets

78 $$ \frac{{\partial \overset{\lower0.5em\hbox{$\smash{\scriptscriptstyle\frown}$}}{u}_{j}^{\left( i \right)} }}{\partial r}|_{r = 1} = \delta_{ij} G_{1} \left( {\alpha ,\beta } \right) + n_{i} n_{j} G_{2} \left( {\alpha ,\beta } \right), $$

where

79 $$ \begin{array}{*{20}c} {G_{1} \left( {\alpha ,\beta } \right) = b\left( {\alpha ,\beta } \right)\left[ {\alpha A^{\prime } \left( \alpha \right) - A\left( \alpha \right)} \right] - d\left( {\alpha ,\beta } \right)\left[ {\alpha aC^{\prime } \left( \alpha \right) - 3C\left( \alpha \right)} \right]} \\ {G_{2} \left( {\alpha ,\beta } \right) = b\left( {\alpha ,\beta } \right)\left[ {\alpha B^{\prime } \left( \alpha \right) - B\left( \alpha \right)} \right] + 3d\left( {\alpha ,\beta } \right)\left[ {\alpha H^{\prime } \left( \alpha \right) - 3H\left( \alpha \right)} \right]} \\ \end{array} , $$

and the primes denote differentiation with respect to the argument. Note that by using Eqs. (77) and (79), one gets $G_{1} \left( {0,0} \right) = - G_{2} \left( {0,0} \right) = - 3/2$ and under Eq. (78), we recover the well-known Stokesian relation [34] for a no-slip spherical particle, i.e. $\partial \hat{u}_{j}^{\left( i \right)} /\partial r = - 3\left( {\delta_{ij} - n_{i} n_{j} } \right)/2$ on *r* = 1.
